# Effect of Electrode Geometry on the Classification Performance of Rapid Evaporative Ionization Mass Spectrometric (REIMS) Bacterial Identification

**DOI:** 10.1007/s13361-017-1818-5

**Published:** 2017-10-16

**Authors:** Zsolt Bodai, Simon Cameron, Frances Bolt, Daniel Simon, Richard Schaffer, Tamas Karancsi, Julia Balog, Tony Rickards, Adam Burke, Kate Hardiman, Julia Abda, Monica Rebec, Zoltan Takats

**Affiliations:** 1 Division of Computational and Systems Medicine, Department of Surgery and Cancer, Imperial College London, London, SW7 2AZ UK; 2 Waters Research Center, 7 Zahony Street, Budapest, 1031 Hungary; 3 Department of Microbiology, Imperial College Healthcare NHS Trust, Charing Cross Hospital, London, W6 8RF UK

**Keywords:** Rapid evaporative ionization mass spectrometry, REIMS, Bacterial identification, Electrode geometry optimization, Monopolar electrod

## Abstract

The recently developed automated, high-throughput monopolar REIMS platform is suited for the identification of clinically important microorganisms. Although already comparable to the previously reported bipolar forceps method, optimization of the geometry of monopolar electrodes, at the heart of the system, holds the most scope for further improvements to be made. For this, sharp tip and round shaped electrodes were optimized to maximize species-level classification accuracy. Following optimization of the distance between the sample contact point and tube inlet with the sharp tip electrodes, the overall cross-validation accuracy improved from 77% to 93% in negative and from 33% to 63% in positive ion detection modes, compared with the original 4 mm distance electrode. As an alternative geometry, round tube shaped electrodes were developed. Geometry optimization of these included hole size, number, and position, which were also required to prevent plate pick-up due to vacuum formation. Additional features, namely a metal “X”-shaped insert and a pin in the middle were included to increase the contact surface with a microbial biomass to maximize aerosol production. Following optimization, cross-validation scores showed improvement in classification accuracy from 77% to 93% in negative and from 33% to 91% in positive ion detection modes. Supervised models were also built, and after the leave 20% out cross-validation, the overall classification accuracy was 98.5% in negative and 99% in positive ion detection modes. This suggests that the new generation of monopolar REIMS electrodes could provide substantially improved species level identification accuracies in both polarity detection modes.

**Electronic supplementary material:**

The online version of this article (10.1007/s13361-017-1818-5) contains supplementary material, which is available to authorized users.

**Figure:**
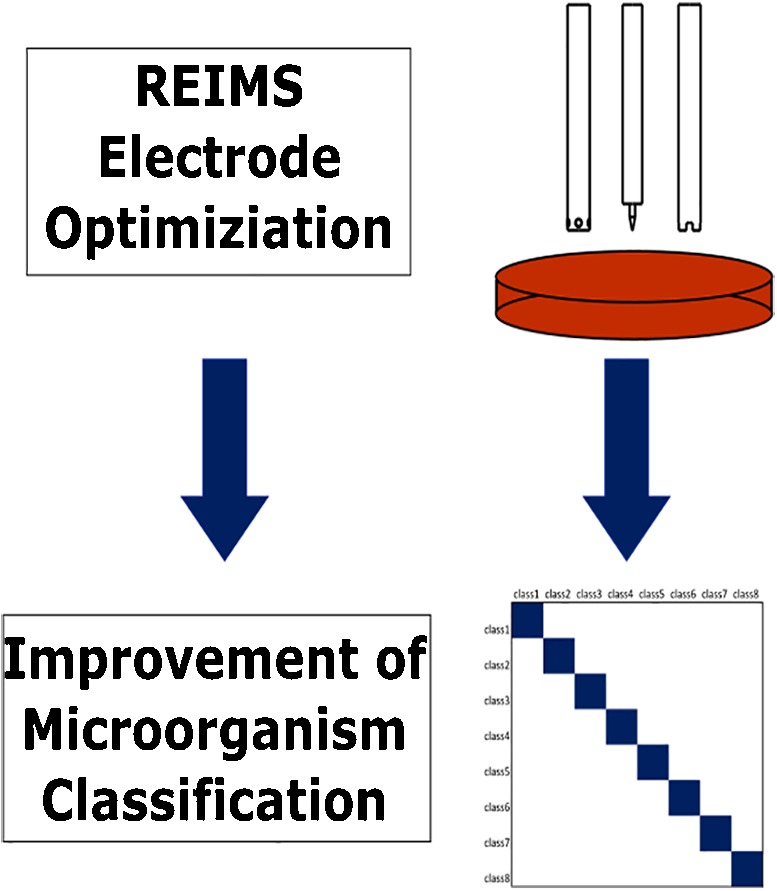
Graphical abstract

## Introduction

Rapid evaporative ionization mass spectrometry (REIMS) is a form of ambient ionization mass spectrometry that requires no sample preparation or extraction before analysis. As a technology, it was primarily developed for the real time identification of tissues during surgical interventions. Coupling mass spectrometry with a standard surgical diathermy device resulted in a technology called the intelligent knife (iKnife). REIMS works by applying a radiofrequency electrical current to a sample biomass, causing it to rapidly heat due to the sample’s non-zero impedance [1]. The resulting thermal disintegration of cells produces an aerosol that is introduced into the REIMS interface of the mass spectrometer for near real-time analysis. The REIMS technology has seen a number of successful applications ranging from the intraoperative, in vivo detection of tumor margins for a diverse range of cancers [2–4], to food analysis of meat products [5], and bacteria and microorganism identification.

The first application of REIMS to the study of microorganisms directly from culture utilized hand-held bipolar forceps similar to those used in brain surgery. Using this REIMS modality, both bacterial and yeast cultures can be analyzed directly from agar plates, without sample preparation or extractions. For analysis, a user picks up a small amount of microbial biomass onto the tip of one of the forceps’ probes, and brings the two probes together initiating a bipolar electrical current between the probes. This causes the sample to heat rapidly and produce an analyte-containing vapor that is aspirated through a small opening in the forceps towards the mass spectrometer via PTFE tubing [6]. Using these bipolar forceps as a REIMS ion source was proven to be a successful method for bacterial classification on 28 clinically important and taxonomically diverse bacterial and yeast species [7].

However, due to the relatively high level of user input needed to operate the hand-held bipolar forceps, this method of REIMS is unsuitable for high-throughput clinical microbiology laboratories, the workflows of which are also becoming increasingly automated [8, 9]. Therefore, development of a solution has been initiated that is more compatible with automated platform requirements. As a starting point, a commercially available workstation was modified to meet the specific REIMS requirements. This automated platform employs monopolar electrodes attached to a robotic arm, instead of the bipolar forceps, for the thermal disintegration of a microbial biomass directly from its culture plate. The robotic system also has an arm for moving the microbial plates, and a camera that can be used for colony selection. Thus, the system requires minimal user input and is well suited to the increasingly automated and high-throughput nature of clinical microbiology laboratories. Importantly, this high-throughput platform was shown to possess the same degree of analytical discrimination as the bipolar forceps REIMS approach, with substantially reduced intra-sample variability [10]. In addition, it was shown to be superior to the hand-held bipolar forceps in the species-level classification of eight *Candida* species, providing perfect accuracy [11].

REIMS provides an alternative to the currently used platforms for microbial identification within clinical microbiology laboratories, which are reliant upon matrix assisted laser desorption ionization time-of-flight mass spectrometry (MALDI-ToF). The primary benefit of REIMS is that it lends itself more easily to integration with automated and high-throughput laboratories [12], due to its minimal user input and automated workflow. Additionally, the workflow for both bacteria and yeast is identical using REIMS, whilst for MALDI-ToF MS-based platforms, the latter requires time consuming and user intensive protein extractions [13]. In contrast, REIMS provides accurate speciation of bacteria and yeasts direct from colonies without the need for any preparative steps. This shows that the REIMS technology is a considerable alternative for the available microbial identification platforms; however, development of the automated REIMS setup is still ongoing to improve classification accuracy.

The aim of this study was to develop and optimize new monopolar electrodes for the high-throughput REIMS platform to achieve better classification of microbial species. For this purpose, different electrode geometries were tested on several clinically relevant bacteria. During the development, optimization decisions were based upon the classification accuracy of bacterial species using principal component analysis (PCA) and linear discriminant analysis (LDA) classification models.

## Experimental

### Identification and Culturing of Bacterial Isolates Used in Optimization Workflow

For the optimization experiments, eight different bacterial species (*Klebsiella pneumoniae*, *Escherichia coli*, *Staphylococcus aureus, Streptococcus pneumoniae, Lactobacillus jensenii, Enterococcus faecium, Enterococcus faecalis,* and *Pseudomonas aeruginosa*) were studied, each with five isolates collected from different clinical samples. All isolates were collected and processed at the Imperial College Healthcare NHS Trust (London, UK) Microbiology Diagnostic Laboratory under standard laboratory protocols. Species level identifications were confirmed using MALDI-ToF-based mass spectrometry as previously described [10]. Bacterial isolates were grown on Columbia blood agar (CBA) at 37 ^o^C in a normal aerobic atmosphere for 24 h. *Streptococcus pneumoniae* and *Lactobacillus jensenii* isolates were cultured in a 5% carbon dioxide atmosphere for 48 h. Composition of CBA is detailed in the Supporting Information.

### Instrumentation

Electrodes were tested with a high throughput colony picker system consisting of a liquid handling robot (EVO 75; Tecan, Switzerland) with an integrated colony picker tool (Pickolo; SciRobotics, Israel). REIMS was implemented by equipping the colony picker head with electric diathermy functionality and connecting it to a Xevo G2-XS Q-ToF (Waters Corporation, Wilmslow, UK) instrument via PTFE tubing. Rapid evaporation ionization was performed using radiofrequency alternating current power supply (470 kHz sinusoid) with an ERBE IC 300 electrosurgical generator (Erbe Elektromedizin, Tübingen, Germany). The vapor produced was co-aspirated with HPLC grade propan-2-ol (ROMIL, UK), which contained 10 μg/mL leucine enkephalin (VWR, UK) as a lock mass reference compound, and set at a flow rate of 0.2 mL/min. Mass spectrometry acquisition was performed in negative and positive ion detection modes, under sensitivity mode within the scan range of 50 to 2500 *m/z* with a 1 s scan time. The ToF analyzer was calibrated on each day of analysis using sodium formate and following the manufacturer’s instructions.

### Construction and Optimization Workflow of Monopolar Electrodes

**Figure 1:**
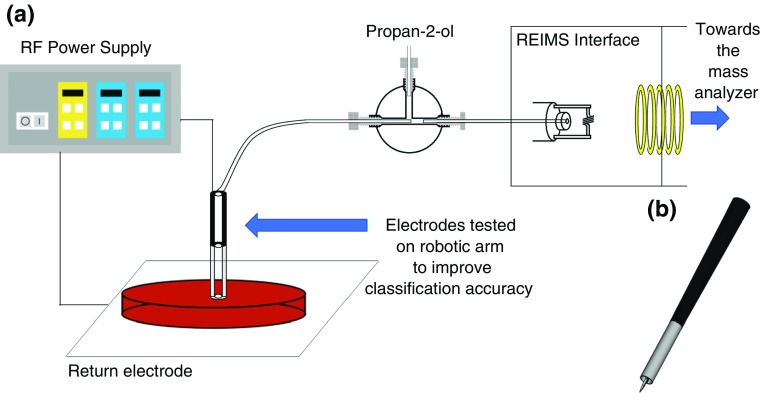
Rapid evaporative ionization mass spectrometry (REIMS) setup with monopolar electrode for bacteria identification **(a)** and a CAD draw of the sharp electrode **(b)**

To produce each bespoke monopolar electrode, 200 μL TECAN tips (TECAN tip type: LiHa, ANSI, pure, non-filtered, conductive, 200 μL, refill insert, part number 30000627) were modified in order to use them as REIMS monopolar electrodes. The top part of the tip remained unchanged to allow the robotic arm to pick up and move the electrodes. The bottom end of the tip was removed and a stainless steel collar was inserted. Figure 1a shows the electrode-mass spectrometry setup (further figures are available in the Electronic Supplementary Material, which show the modifications of the electrodes) After preliminary experiments with different designs of electrodes, the best signal-to-noise ratio and classification accuracy was achieved using sharp point and round-tube shape geometries. Thus, detailed optimization was performed on these geometries. Owing to the differences observed between bacterial species, in regards to optimal values for tip length and heating power, measuring optimization success through mass spectral signal intensities and signal-to-noise ratios was not productive. In order to overcome this problem, a different method was chosen to find the optimal geometry. REIMS technology has proven to be excellent for tissue and bacteria classification/identification ability; thus, optimization was performed via multivariate statistical models and cross-validation. However, to achieve good and reliable results, a high number of samples and experiments were necessary. For each electrode, each of the five isolates from each of the eight bacterial species used were analyzed with five different sampling points. In the case of low biomass producing bacteria (e.g., *Lactobacillus jensenii*) sampling was performed on a single colony. The electrodes were cleaned between samples using propan-2-ol.

**Figure 2:**
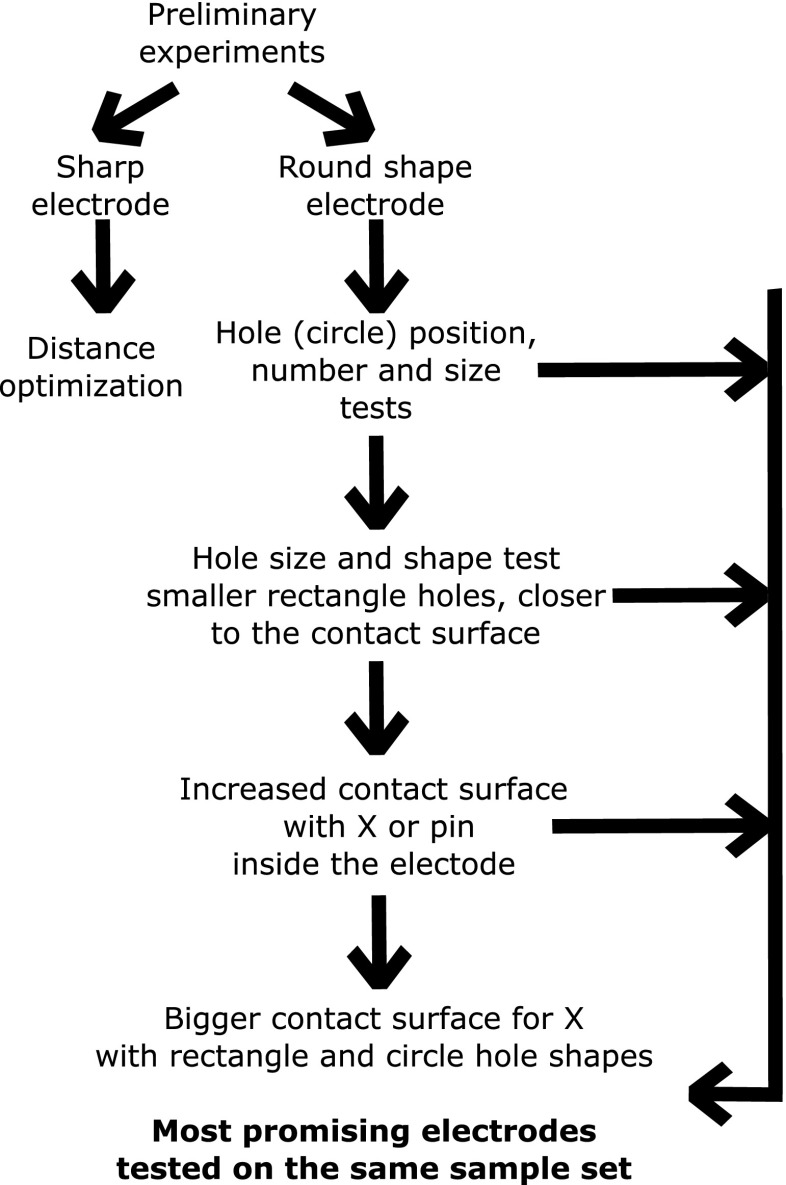
Optimization workflow to decide which electrode gave the highest classification accuracy. Round shaped electrodes were optimized in separate batches; then the best electrodes were tested on the same bacteria colonies for final decision

Optimization by classification accuracy was tested with the sharp electrodes. To test the robustness of the results, data were collected in both positive and negative ion mode. Optimization of the sharp point electrodes focussed on the distance between the contact point and the entrance to the electrode tubing. A total of five different distances were tested: 0.5, 1.0, 2.0, 3.0, and 4.0 mm. A piece of rubber tubing was used to shorten the distance using the standard 4.0 mm probe. After these distance experiments, the same isolates were prepared twice more and analyzed in the same way in negative ion mode to test the reproducibility of the classification accuracies. Based on these results, further optimization was performed in batches and the best electrodes were selected and tested together on the same grown bacteria colonies. Figure 2 details the optimization workflow followed during testing of both sharp and round shaped electrodes.

For the round shaped electrodes, a number of prototype geometries were tested. In order to avoid picking up the plate (due to the lower pressure in the mass spectrometry tube), holes were drilled into the wall of each electrode (El1, El2, El3, El4, El5). Additionally, these holes may have improved the sampling efficiency through aspiration of additional analyte-containing vapor from outside of the electrode. Therefore, different hole shapes – rectangle (N1, N2, N3, N4) and circle (El1, El2, El3, El4, El5) – , sizes, and positions were tested, as detailed in Table 1. Additional features were also added to the round shaped electrode (detailed in Table 1.), such as a metal “X” -shaped insert of varying size (X1, X2, X3, X4, X5, XC1, XC2) and a pin in the middle (XB), to increase the contact surface. Technical drawing of each electrode is shown in Electronic Supplementary Material. Sharp electrode 3D CAD draw is shown as example of the electrodes on Figure 1b.

**Table 1:** Detailed Information of the Tested Electrodes

ID	Shape	Hole on the wall of the electrode				Insert (diameter of pin/mm or width of X/mm)	Heating power (W)	PCA classification accuracy (%)	
		Closest distance from the contact surface /mm	Shape	Number	Size (diameter/mm or width x height/mm)			Negative ion mode	Positive ion mode
Original ^a^	sharp	no holes	no holes	no holes	no holes	no insert	17	77/80/87	33
3 mm ^b^	sharp	no holes	no holes	no holes	no holes	no insert	17	82	42
2 mm ^b^	sharp	no holes	no holes	no holes	no holes	no insert	17	93	61
1 mm ^b^	sharp	no holes	no holes	no holes	no holes	no insert	17	89	63
0.5 mm ^b^	sharp	no holes	no holes	no holes	no holes	no insert	17	90	49
EL1	round	0.5 mm	circle	5	1	no insert	40	86	60.5
EL2	round	0.5	circle	3	1	no insert	40	92.5/90.5	66.5/91
EL3	round	0.5	circle	3	1.5	no insert	40	86.5	52
EL4	round	2.5	circle	3	1	no insert	40	91	52
EL5	round	0	half circle	6	1	no insert	50	92/89	76.5/77
N1	round	0	rectangle	4	0.5 × 0.5	no insert	50	83	78.5
N2	round	0	rectangle	4	1 × 0.5	no insert	50	83	76.5
N3	round	0	rectangle	4	0.5 × 1	no insert	50	84.5/93	80/85.5
N4	round	0	rectangle	4	1 × 1	no insert	50	86	76.5
X1	round	0.5	circle	2	1	0.25 X	50	81.5	83
X2	round	0.5	circle	2	1	0.5 X	50	87/93.5	80.5/89
X3 ^c^	round	no holes	no holes	no holes	no holes	0.5 X	30	83	59.5
XB	round	0.5	circle	2	1	0.5 pin	50	83.5	74.5
X4	round	0.5	circle	2	1	1 X	80	88	82.5
X5	round	0.5	circle	2	1	2X	110	77	58.5
XC1	round	0	rectangle	4	0.5 × 0.5	1 X	100	86.5	84
XC2	round	0	rectangle	4	0.5 × 0.5	2X	110	79.5	72

^a^Distance between the contact point and the entrance to electrode tubing is 4 mm.

^b^Distance between the contact point and the entrance to electrode tubing.

^c^The cross was raised 0.1 mm from the pa.

Optimal heating powers were determined for each of the electrodes on the same isolate of *E. coli* and *S. pneumoniae*, with three sampling points, before analysis of all isolates from eight bacterial species. *E. coli* and *S. pneumoniae* were chosen for the heating power tests as the appearance and colony size, as well as the signal and signal-to-noise ratio, show significant differences between the two species. The heating power was increased in 3 W intervals from 2 to 40 W for each of the sharp electrodes, and for the round shape electrodes optimization begun at 10 W, increased by 10 W increments up to 160 W. Optimal heating power was determined based on signal intensities and signal-to-noise ratios. The heating power used for the sampling is shown in Table 1 for each electrode.

### Mass Spectral Processing and Data Analysis

The Offline Model Builder (ver. 1.1.29.0) software (Waters Research Center, Budapest, Hungary) was used for mass spectral processing, including background subtraction, mass drift correction, total ion count normalization, and mass binning to 0.1 Da. Data was exported from OMB into csv file for data analysis of the background subtracted signal and signal-to-noise ratio. Signal was defined as a high intensity peak (bin) for each bacterium – 733.5 for *Escherichia coli* and 925.6 for *Streptococcus pneumoniae*. Noise was defined as the sum of the bin intensities between the 600 and 610 region where no bacteria-related peaks were detected. Signal-to-noise ratio was calculated for each sampling point using the selected bacteria bin and the summed noise.

As the primary purpose of monopolar electrode probe optimization was to maximize species-level classification accuracy, it was decided to measure the success of optimization using this metric. In order to improve classification accuracy of the REIMS platform, each group of electrodes was tested with multivariate statistical approaches using principal component (PCA) analysis and linear discriminant analysis (LDA). Classification models were built in Offline Model Builder software using the 600–1000 *m/z* range in negative ion detection mode, and 400–1000 *m/z* range in positive ion detection mode. For the construction of principal component analysis classification models [14], a total of 50 components were used, and for linear discriminant analysis classifications models [15], a total of eight dimensions were used, after reduction of the dimensionality of the data using PCA. Classification models were tested using a cross-validation whereby 20% of each sample class was removed and the remaining 80% of each class used to construct a classification model. The withheld samples were then classified based on the distance to the closest class in the PCA/LDA space based on the Mahalanobis distances between objects [16]. During the optimization workflow, PCA classification models were used for comparisons between electrodes as their unsupervised approach allows for a greater degree of potential differences between electrodes to be observed and because, comparered with the LDA model, the PCA model also can give information about noise of the measurments. A high number of PCA dimensions were used to explain the highest variance of the data and eight LDA dimensions were used since eight groups were analyzed. Using too many principal components can easily result in overfitting the model and could lead to false conclusions. In order to confirm the conclusion, a different model was also built in Matlab (R2016a). In Matlab, maximum margin criterion linear discriminants analysis (MMC LDA) was used as a supervised classification method. To prevent overfitting, a leave-plate-out cross-validation scheme was used, whereby observations from each plate were projected onto a model determined with observations from all other plates. Observations from the omitted group were classified according to the shortest distance to the class centroids. Confusion matrices were determined showing the classification and overall accuracy. Dimensionality reduction is required before MMC LDA, with the number of PC dimensions used being limited to one less than the number of classes (minumum of 2).

### Safety Considerations

In this work, all microorganisms were treated as Hazard Group 2 organisms and were therefore manipulated within containment level 2 facilities. All cultures and REIMS analyses were performed within a class 2 biological safety cabinet. All solvents, such as propan-2-ol, were handled according to the material safety data sheet provided by their respective manufacturer.

## Results and Discussion

**Figure 3:**
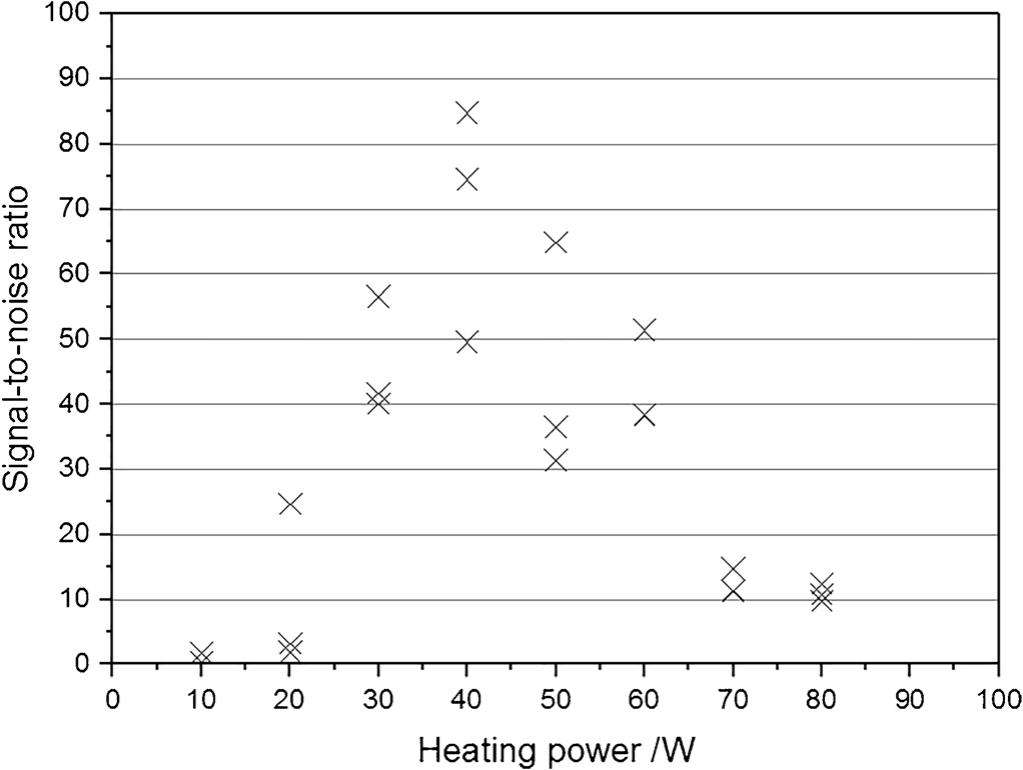
Signal-to-noise ratio with different heating power in the case of *Streptococcus pneumoniae* with EL5 in negative ion detection mode. Optimal heating powers were selected for the heating power that gave the maximum signal-to-noise or where a plateau had been reached

Experiments were started with the optimization of the heating power of each electrode. Each incremental increase in heating power provided higher signal intensities; however, the background signal also increased. Electrodes with a small contact surface (e.g., sharp electrodes) required lower heating power to provide an intense phospholipid signal (600 to 1000 *m/z*), whilst a higher heating power was needed for electrodes with larger contact area. These results were in agreement with the expectations and in accordance with the principle that the same heating power applied to a larger surface area will result in a lower evaporation temperature. If the temperature is too low, water evaporates slowly, giving time for carbonization. In contrast to this, higher temperature causes rapid disintegration of the bulk phase of the sample producing fine aqueous aerosol in case of high water content biological samples. An example of the heating power effect on the signal-to-noise ratio and intensities is shown by Figure 3. For all tested electrodes, the signal-to-noise ratio either reached a maximum and then plateaued as the heating power was increased, or reached a maximum and then began to decrease. Optimal heating powers were selected where the signal-to-noise was at a maximum, or where a plateau had been reached. The same optimal values were found for both kinds of bacteria (*E. coli* and *S. pneumoniae*) tested with the same electrodes. These selected values were used in further experiments and are summarized in Table 1.

**Figure 4:**
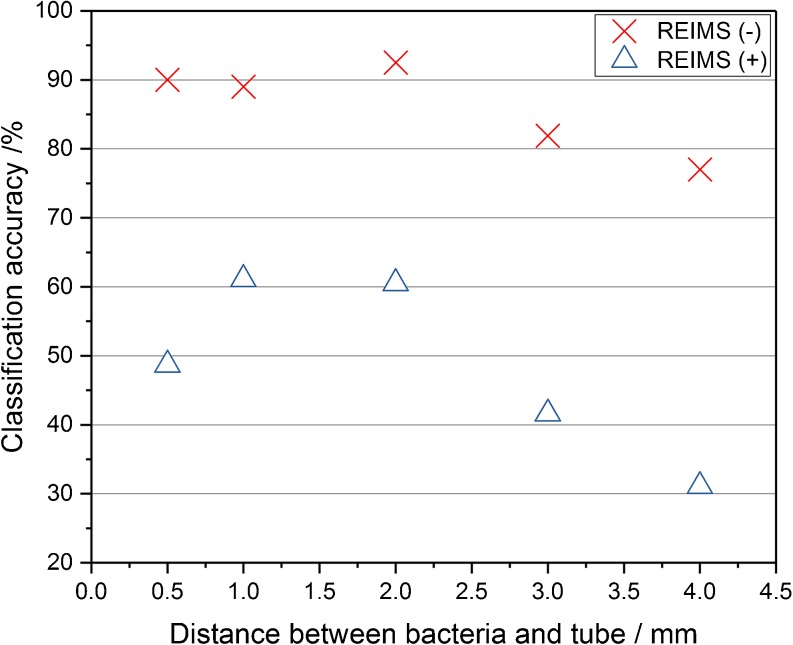
Classification accuracy results in negative and positive ion mode with different distance between the contact point and inlet to the sharp electrode probe. Long distance resulted in less precise classification accuracy

The sharp tip monopolar electrodes have better spatial resolution, due to the reduced size of the contact surface. Therefore they can be used when accurate positioning is required, such as for the analysis of microorganisms that grow as small colony sizes or for imaging of bacteria colonies and tissue [17]. For optimization of the sharp tip electrodes, the distance between the contact point and inlet to the electrode probe was altered and tested. Absolute intensities, TIC normalized intensities, and signal-to-noise ratio were tested. During the acquisition and data analysis, we felt that intensities were higher and it was easier to detect the sampling points with short distance electrodes; however, it was difficult to conclude which setting gave the best results based on the intensities and signal-to-noise ratio. Different bacteria have different optimal distances and these intensity related values also suggested different optimal settings sometimes also overwhelmed by poorer reproducibly than the difference between the tested setups. Examples of these challenging result sets are shown in the Electronic Supporting Information for *Escherichia coli, Lactobacillus jensenii*, and *Streptococcus pneumoniae*. As the primary goal of this research was to improve species-level classification accuracy of the platform, leave 20% out cross-validation was used to test classification accuracies for multivariate statistical models. Shorter distance improved the species-level classification accuracy rates: cross-validation values were increased by reducing the distance to 2 mm (Figure 4) to 91% in negative and 63% in positive ion detection modes. Classification accuracies remained the same with both 1.0 mm and 0.5 mm distances in negative ion detection mode. Similar results were given with the MMC leave one plate out cross-validation method (results are shown in the Supporting Information Table 1.). In positive ion detection mode, the shortest distance, 0.5 mm, provided slightly reduced classification accuracies compared with 1.0 or 2.0 mm distances. This may have been the result of the rubber tubing used to shorten the distance between the contact surface and electrode inlet, making contact with the bacterial surface and thus impeding the electrical current and limiting the heat dissipated within the bacterial biomass. As the classification accuracies did not improve with shortening the distance, 2.0 mm was selected as optimal for the sharp tip electrode. For future designs of sharp electrodes, the rubber part will be omitted and electrodes will be produced with a shorter pin/closer inlet tube.

These results showed that a sufficient number of sampling points were used to achieve reliable optimization results via classification accuracies. All these data were collected on the same day, with the same batch of bacteria; therefore, it was also necessary to test reproducibility on fresh grown bacteria colonies. The aim of this reproducibility study was to investigate if electrodes, initially tested on different batches of bacteria, have to be tested together again on the same batch to conclude which electrode gives the best classification accuracy. For that purpose, bacteria were grown again two more times and the 4.0 mm electrode was tested in negative ion mode. The following classification accuracies were calculated for the three batches: 77%, 80%, 87% (RSD = 5.2%). This RSD value seems acceptable; however, the difference between the values suggests that an additional step would be helpful to finalize the optimization results. Therefore, instead of increasing the number of isolates for the round shape electrodes, the best electrodes from each batch were then tested together on a single batch of bacteria in a last round to determine which gives the highest classification accuracy.

Owing to their larger contact surface, round shape electrodes could potentially evaporate more microbial biomass from one sampling point, which would be particularly beneficial to species that produce low biomass volumes during standard, pure growth on agar plates. However, in the case of mixed cultures, accurate sampling and bacteria identification could be challenging. As a result of the entire contact surface area of the round shape electrodes making contact with the sample, a sealed vacuum is created resulting in the picking up of the agar plate. To solve this problem, small holes were drilled into the sides of each electrode to allow a break in the vacuum to be created. Additionally, we hypothesized that these holes may also improve the sampling efficiency, through the aspiration of a greater volume of analyte-containing vapor. Therefore, the first step in the optimization of round shape electrodes investigated the effect of the position and size of holes. Overall classification accuracies were improved by 6.5% in negative (increased to 92.5%) and 6% in positive ion detection modes (increased to 66.5%) with three 1 mm holes (EL2) compared with five 1 mm holes (EL1). Classification accuracies are summarized in Table 1. When three 1.5 mm holes (EL3) were used instead of the 1 mm holes, the overall classification accuracies decreased by 6% in negative and 14.5% in positive ion detection modes, compared with EL2. A likely source of this decrease is that the evacuation of the analyte-containing vapor is less effective and the vapor is also diluted with sample-free air, thus the mass spectral data produced is not a true representation of that which has been produced during the REIMS process. As a next step, the positioning of the holes was investigated. Three distances were tested (2.5, 0.5, and 0.0 mm from the contact surface) and the overall classification accuracies were 52%, 66.5%, and 76.5%, respectively, for EL4, EL2, and EL5 in positive ion detection mode. This suggests a negative correlation between distance and cross-validation accuracy. In negative ion detection mode, the classification accuracies were similar (91% to 92.5%) even when the hole was 3 mm away (EL4) from the contact surface. This meant classification accuracy was improved or unchanged with the shorter distance. Therefore, the following generation of electrodes had the holes as close to the contact surface as possible, and they only had two 1 mm holes when it was necessary in order to avoid plate pick up.

Further optimization of the sampling hole position and sizes was continued. A set of new electrodes was produced that contained rectangle shaped holes towards the contact surface end of the electrodes with different hole sizes. Overall, classification accuracies showed minimal change as a result of this optimization. In negative ion detection mode, an improvement from 83% to 86% was observed, and in positive ion detection mode, an improvement from 76.5% to 80%. These results suggest that the hole size should be smaller than three 1 mm holes, but, crucially, that this parameter is not a central component for optimization of the monopolar round shape electrode.

Electrodes with increased contact surface, created through the insertion of metal shapes at the contact surface at the base of the round shape electrodes, were investigated to explore further electrode optimization. In negative ion detection mode, the largest contact surface inside the tube (X2) showed the greatest degree of improvement in classification accuracy (87%); however, in positive ion detection mode, the thinner “X” shape insert (X1) showed the greatest improvement in cross-validation accuracy (83%). The remaining two electrodes (X3 and XB) showed reduced classification accuracy, and a substantial increase in the signal-to-noise ratio, which made it difficult to detect each sampling point on the total ion current (TIC) plot. However, it is important to note that the round shape electrode containing the thinnest “X”-shaped insert (X1) was very fragile and hard to clean, which is necessary to prevent carryover. As the intention of the optimized monopolar electrodes is to improve the application of the automated and high-throughput REIMS platform to clinical microbiology laboratories, this difficulty in cleaning is likely to prevent the adoption of the X1 electrode.

**Figure 5:**
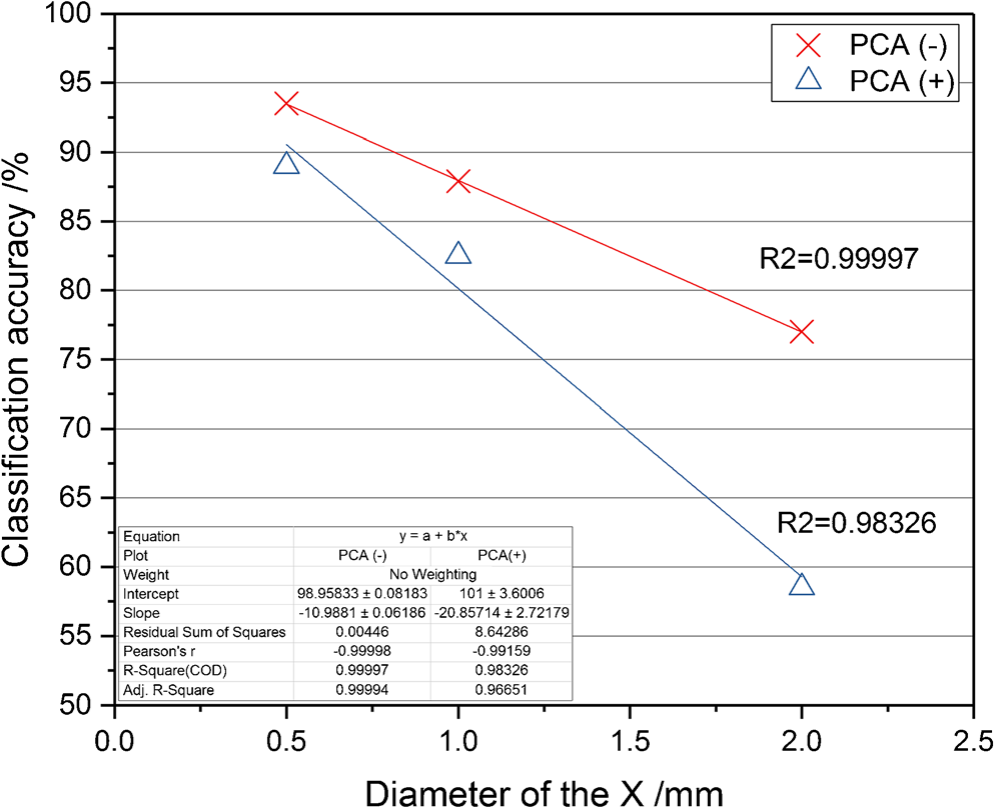
Classification accuracy results in negative and positive ion mode with different diameter of X insert inside the electrode. Increased contact surface also meant smaller inlet space, thus, aspiration of analyte containing vapor was less effective and classification accuracy was lower

Further optimization experiments were carried out with four original prototype electrodes (EL2, EL5, N3, and X2), and four new prototype electrodes (X4, X5, XC1, and XC2) developed based on the findings of previous optimization experiments, to determine the effect of increased size of the contact surface electrodes. As shown by Figure 5, increasing the surface area did not result in an improvement in the classification accuracies. This can be explained by the decrease of gas conductance with the smaller inlet space provided in the round shape electrode preventing the effective analyte-containing vapor from being aspirated. Plotting of the classification accuracies against the size of the “X” contact surface insert, Figure 5, shows a negative linear regression between the two variables; therefore, further increase of the contact surface inside the electrode is not favorable.

On the last test set of electrodes, the highest cross-validation accuracies were achieved with the previous generation of prototype electrodes (EL2, EL5, N3, and X2). This suggests that the size of the hole on the side of the collar of each electrode does not have a substantial impact on sampling efficiency, and serves the primary purpose of preventing the creation of vacuum and picking up of the agar plate being sampled. In negative ion detection mode, the classification accuracy scores ranged between 89% (EL5) and 93.5% (X2), and in positive ion detection mode it was between 77% (EL5) and 91% (EL2). However, there was no optimized electrode that provided a substantially superior cross-validation accuracy, and thus a supervised LDA classification model was built to maximize species-level classification accuracy. As with PCA classification models, a leave 20% cross-validation model was created, which produced classification accuracies between 97.5% (N3) and 99% (EL5 and X2) in negative ion detection mode and between 95% (X2) and 99% (EL2) in positive ion detection mode.

Although substantial improvements in cross-validation accuracy were achieved through optimization of the monopolar electrodes, the overall difference between the four best electrodes (EL2, EL5, N3, and X2) was not significant. Therefore, in choosing the optimal electrode and analytical setup, consideration was also given to the ease of production of each electrode. For this reason, the electrodes containing “X”-shaped contact surfaces were dismissed as the difficulty in production and cleaning would make them impractical for high-throughput clinical microbiology work. Therefore, for future experimental work, the round shape electrode with two 1 mm holes placed on the sides will be used when probe contact with a large contact surface is feasible. However, for samples with low biomass, such as slow growing and/or fastidious microorganisms, the shortened sharp tip electrode with a 2.0 mm distance between the contact surface and probe inlet will be used.

## Conclusions

We have performed detailed geometry optimization of monopolar REIMS electrodes to improve species-level identification accuracy using the automated and high-throughput monopolar REIMS platform. The system described is capable of high accuracy microbial species identification; however, there is still scope to further improve accuracy, particularly for microorganisms that produce low volumes of biomass during growth.

As expected, electrode geometry had a large impact on species-level classification accuracy. Not only did negative mode results improve greatly, but data collected in positive mode also gave excellent species-level classification. As both negative and positive data gave good classification accuracy, there is potential for both ion modes to be used to achieve even better species-level classification accuracies in the future. Further tests using different geometries could also enable a better understanding of the REIMS ionization mechanism, and the effect of current density and gas conductance on the signal and classification. The novel electrodes developed could have numerous applications: sharp electrodes could be used not only for slow growing low biomass bacteria but also for imaging or testing bacteria colonies’ interactions. When spatially resolved analysis is not needed, a larger contact surface could be used to increase the volume of aerosol and therefore increase the signal-to-noise ratio for the tested sample. An increase in signal can be useful when poor signal is acquired with the sharp electrode or when a low limit of detection is needed. The developed electrode can also be used with the robotic platform for other applications, from high-throughput expansion of the iKnife database with spatially resolved data to high-throughput analytical testing of food.

## Electronic Supplementary Material

ESM 1 (DOCX 2991 kb)
