# Supplementary material for: Effect of Electrode Geometry on the Classification Performance of Rapid Evaporative Ionization Mass Spectrometric (REIMS) Bacterial Identification
Source: J Am Soc Mass Spectrom. 2017 Oct 16;29(1):26–33. doi: 10.1007/s13361-017-1818-5 (PMC5785610; doi:10.1007/s13361-017-1818-5)
Supplement: Supplementary file 1 — (DOCX 2991 kb) [file 13361_2017_1818_MOESM1_ESM.docx]

**Electronic supplementary material for**

**Effect of Electrode Geometry on the Classification Performance of Rapid Evaporative Ionisation Mass Spectrometric(REIMS) Bacterial Identification**

**Short Title:** Geometry optimization of REIMS electrodes

Zsolt Bodai (1)*, Simon Cameron (1), Frances Bolt (1), Daniel Simon (2), Richard Schaffer (2), Tamas Karancsi (2), Julia Balog (2), Tony Rickards (1,3), Adam Burke (1), Kate Hardiman (1), Julia Abda (1), Monica Rebec (3), Zoltan Takats (1)

(1) Section of Computational and Systems Medicine, Department of Surgery and Cancer, Imperial College London, London, SW7 2AZ, United Kingdom;

(2) Waters Research Centre, 7 Zahony Street, Budapest, 1031, Hungary;

(3) Department of Microbiology, Imperial College Healthcare NHS Trust, Charing Cross Hospital, London, W6 8RF, United Kingdom

* Corresponding Author: Zsolt Bodai, PhD, Division of Computational and Systems Medicine, Department of Surgery and Cancer, Imperial College London, South Kensington Campus, Sir Alexander Fleming Building, London, SW7 2AZ. Telephone: 020 7594 2760. Email: z.bodai@imperial.ac.uk

**Composition of Columbia Blood Agar (PB0122A)**

Columbia Blood Agar Base (special peptone 25 g/l, starch 1 g/l, Sodium chloride 5 g/l, agar 10.25 g/l) was suspended in de-ionised water. It was sterilised at 121°C for 15 minutes. After the sterilisation, it was cooled and aseptically defibrinated horse blood (50 millilitres / litre) was added.

**Electronic supplementary material Figure 1. Modification of TECAN tips to form electrodes**:

2 cm was removed from the end of the pure TECAN tip and the 4 cm long electrode was inserted tightly into the tip as show in figure below.


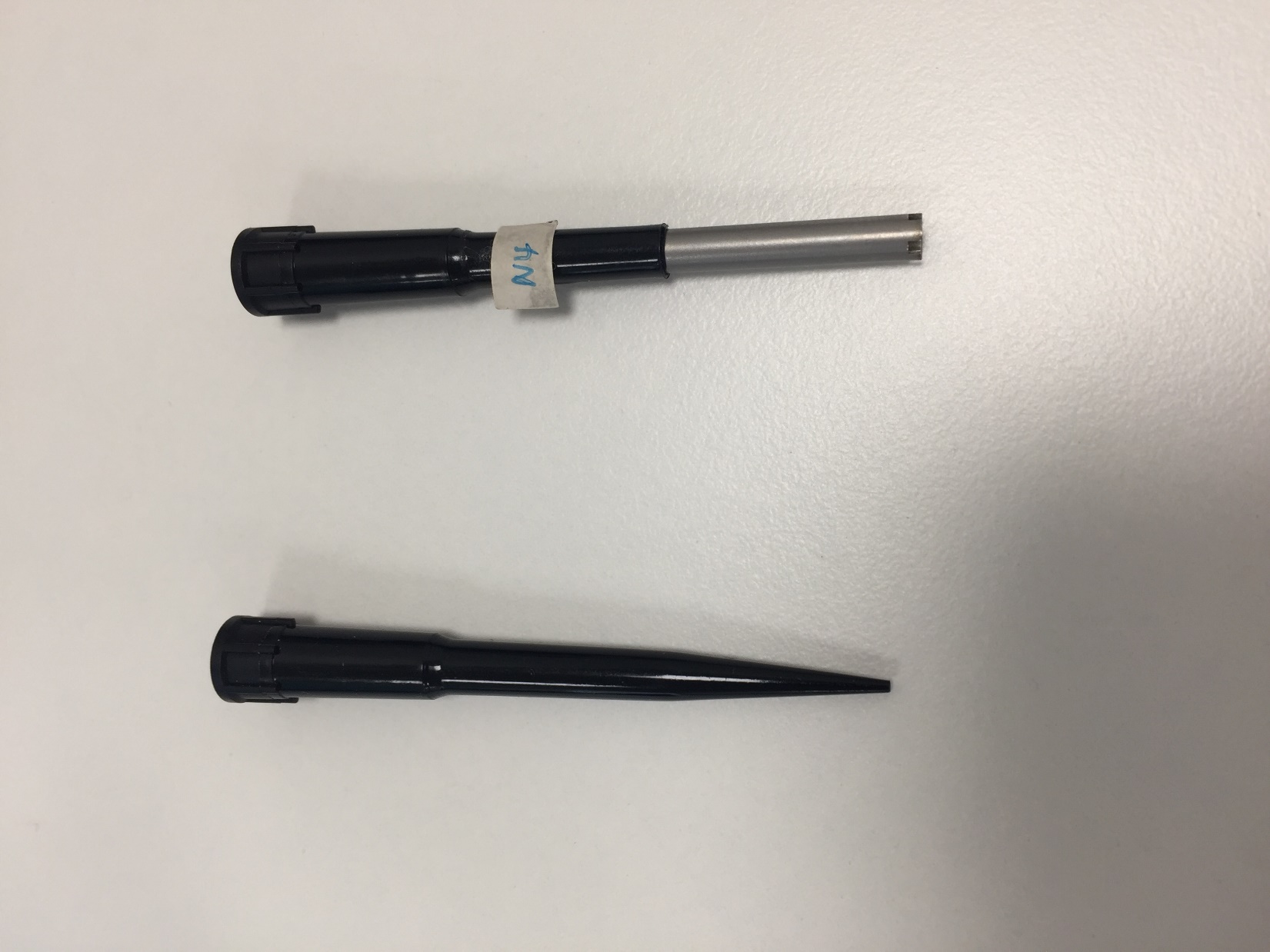


Neat TECAN tip

N4 electrode


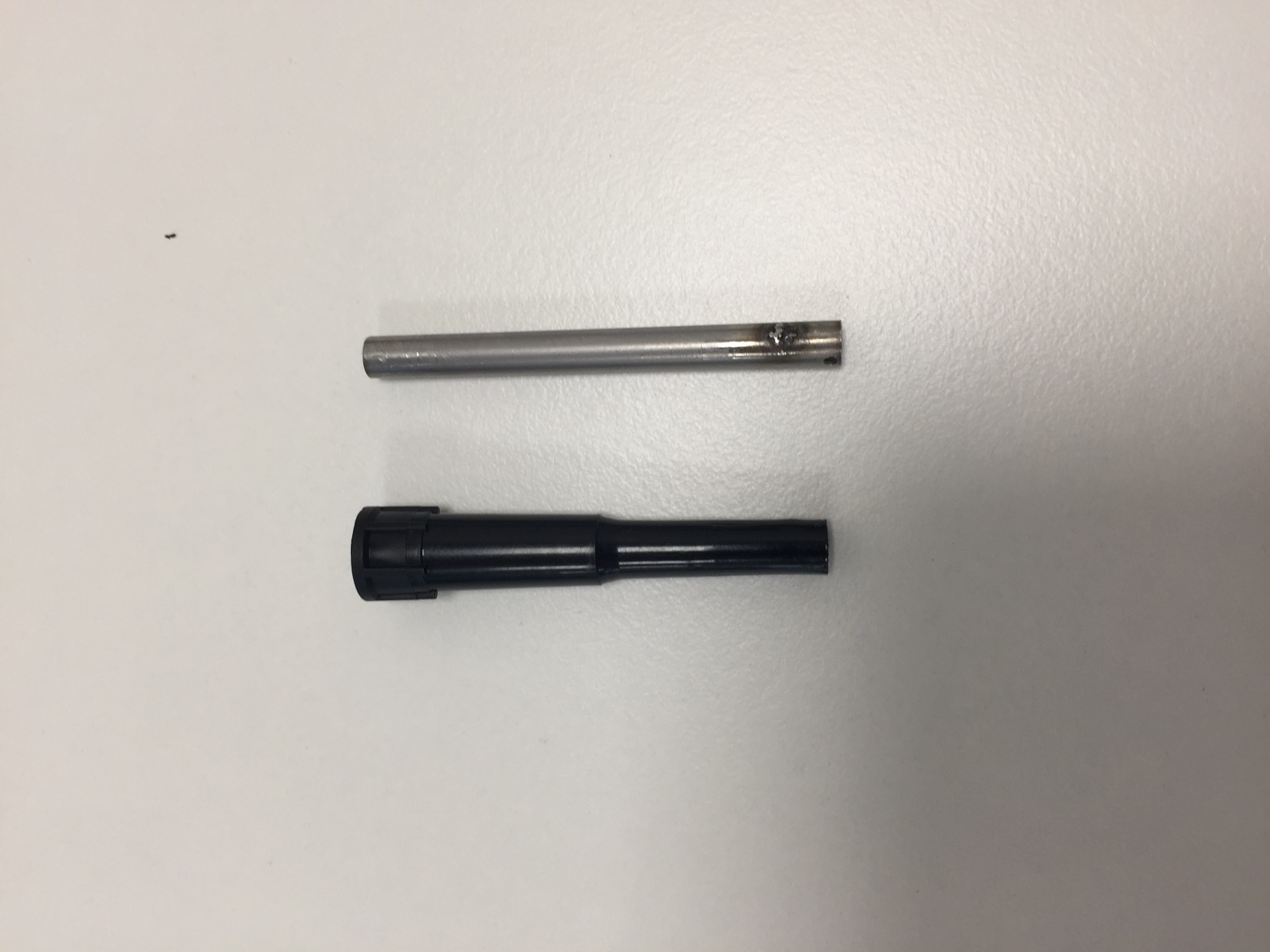


Cut TECAN tip

Neat electrode

**Electronic supplementary material Figure 2. Technical drawing of each electrode:**

Basic electrode


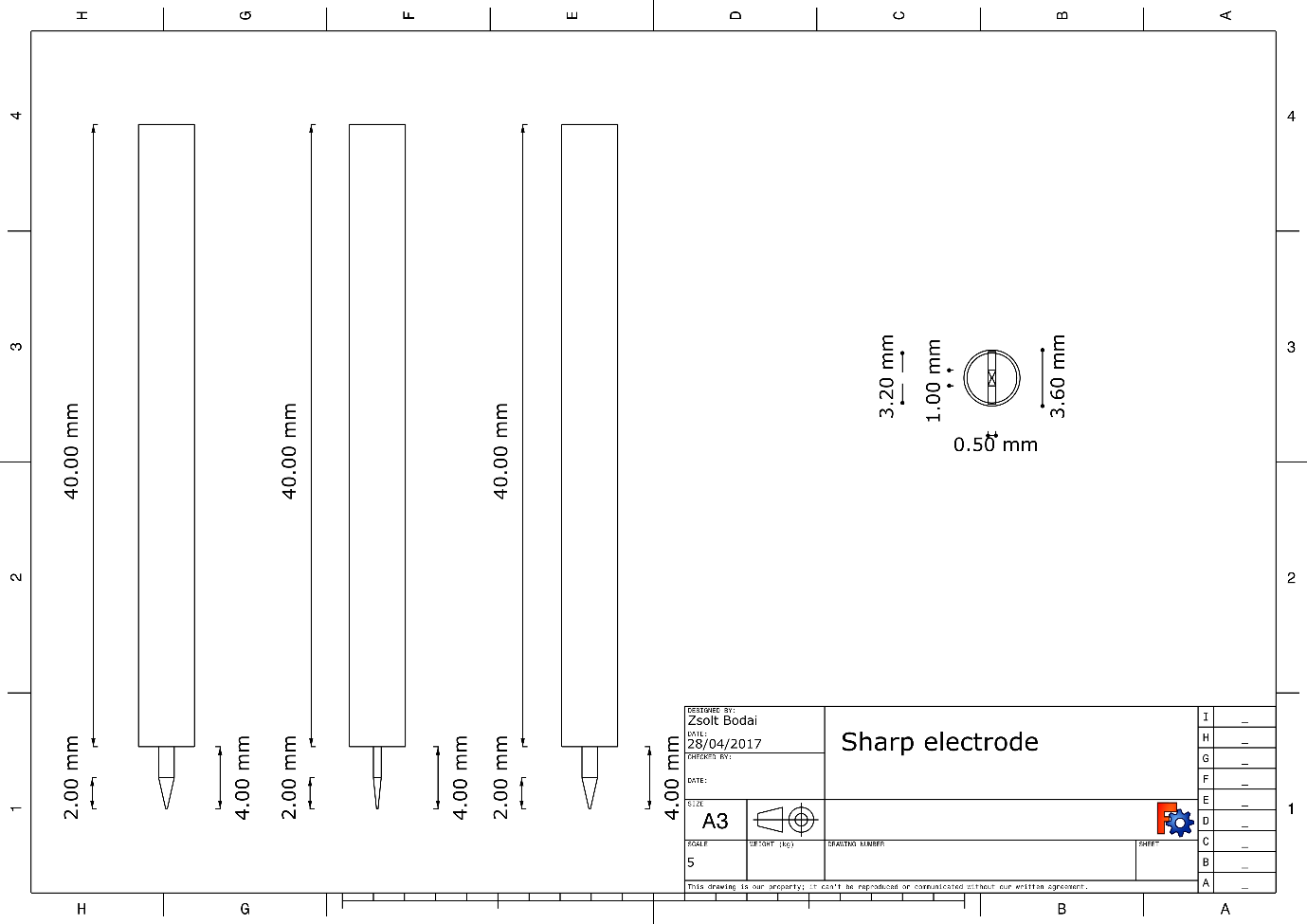


El1 Electrode


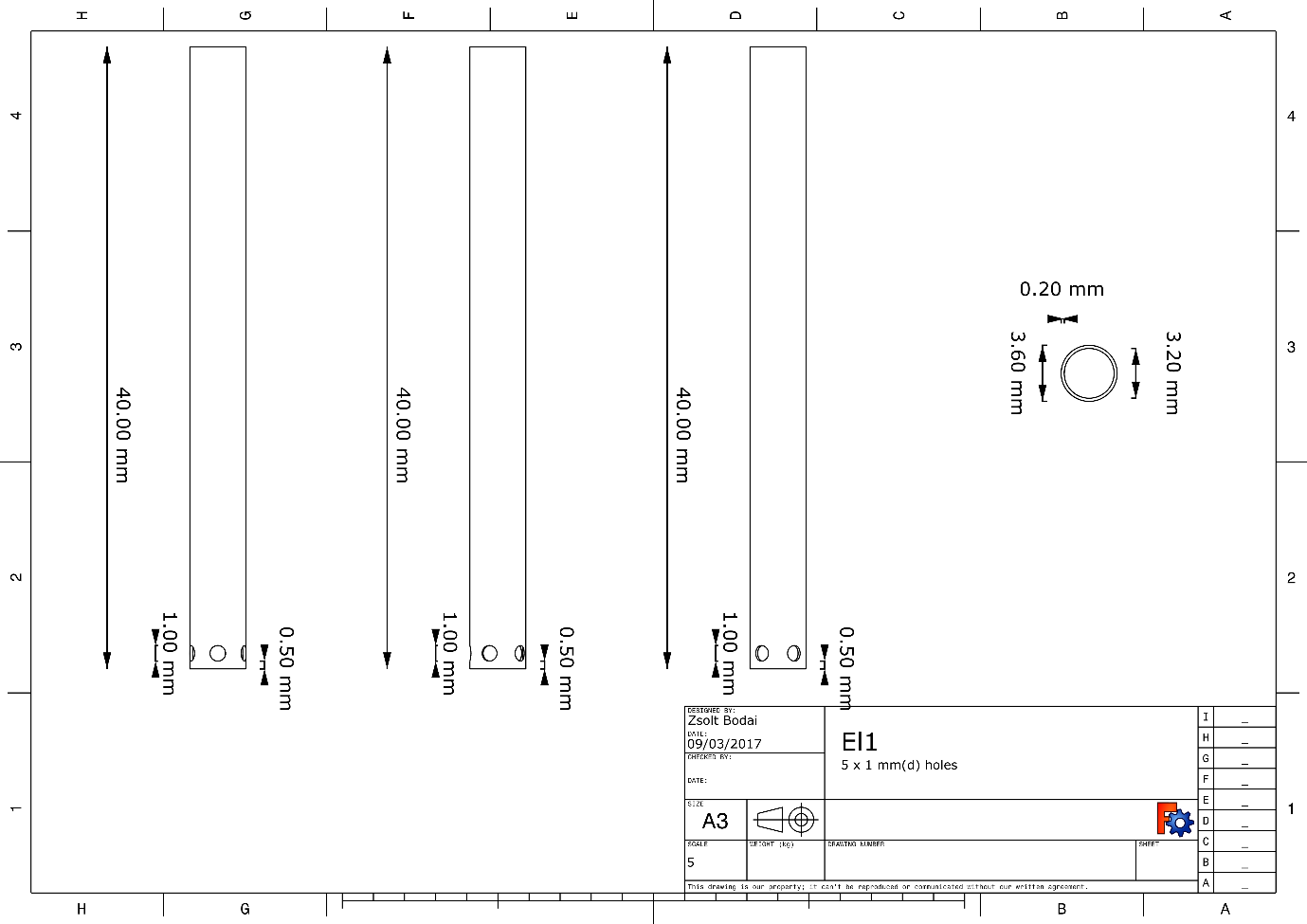


El2 electrode


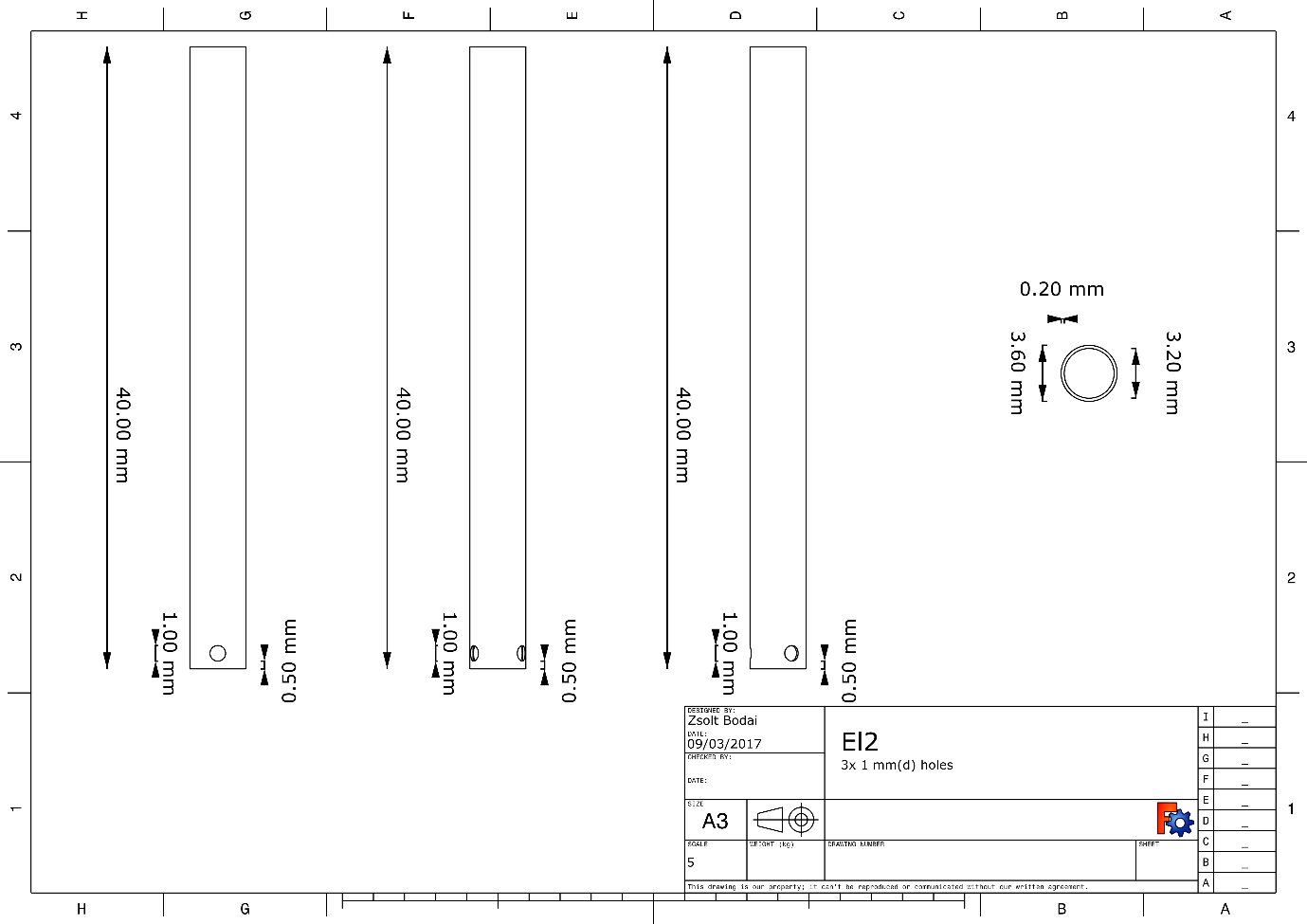


El3 electrode


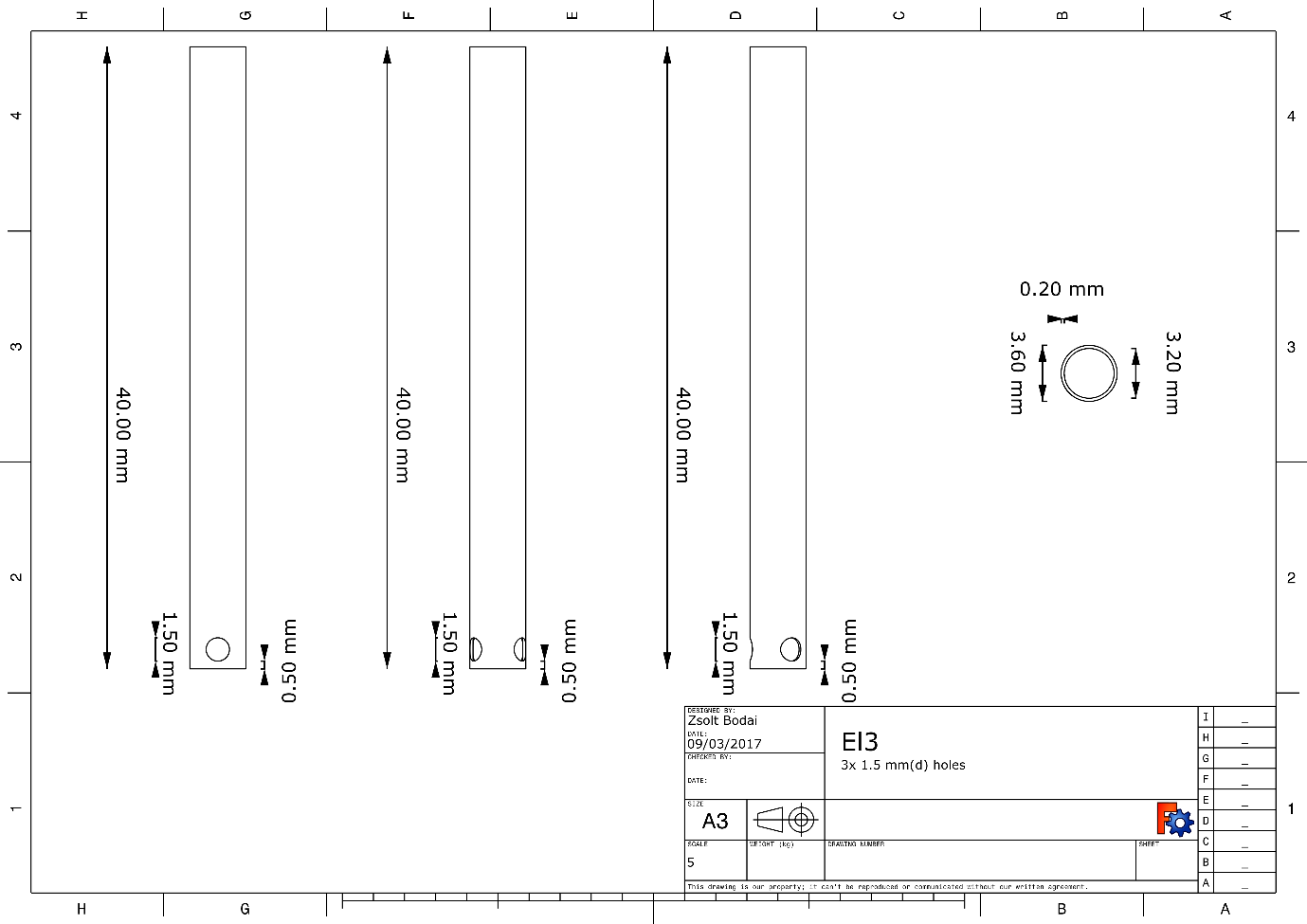


El4 electrode


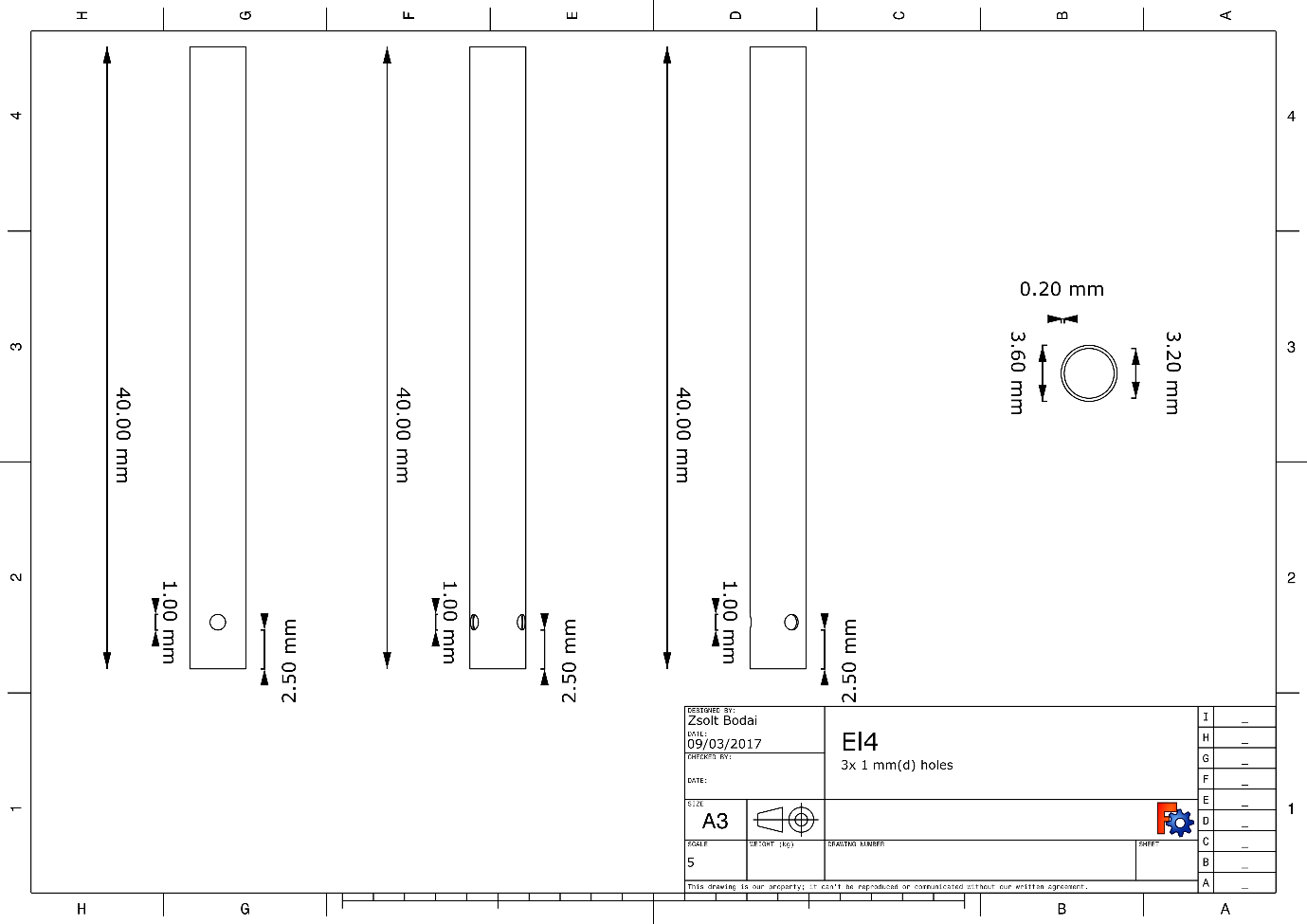


El5 electrode


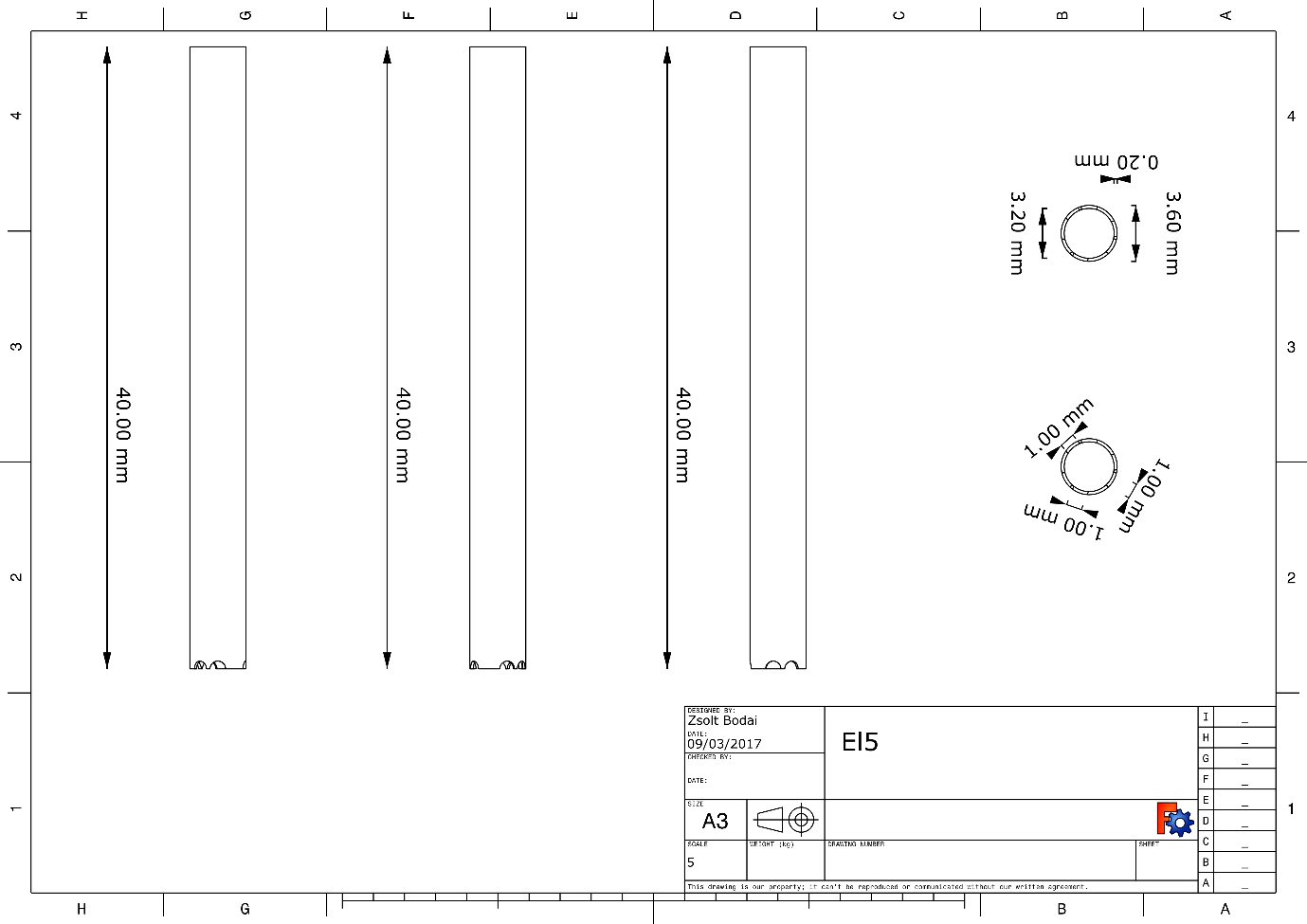


N1 electrode


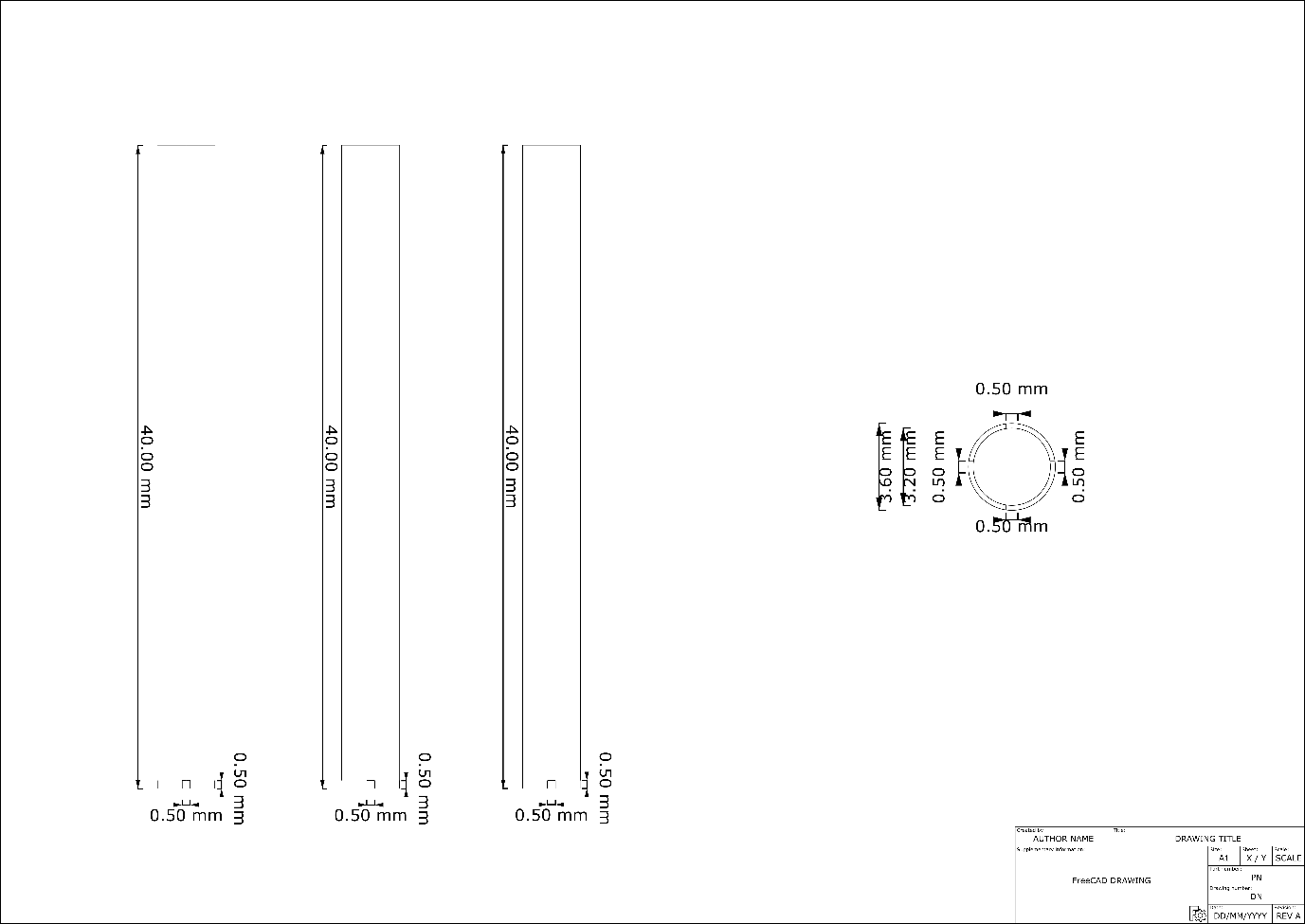


N2 electrode


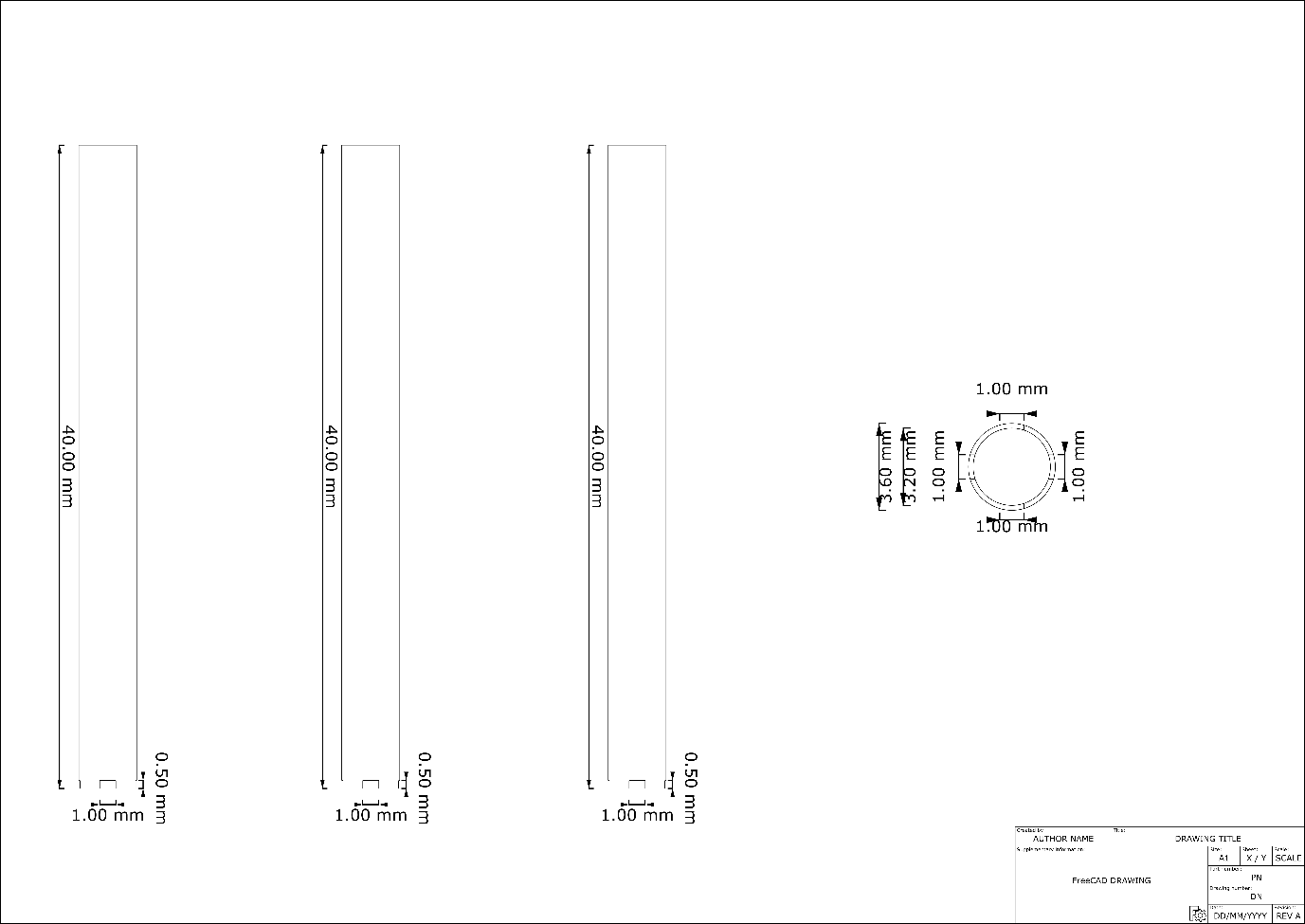
N3 electrode


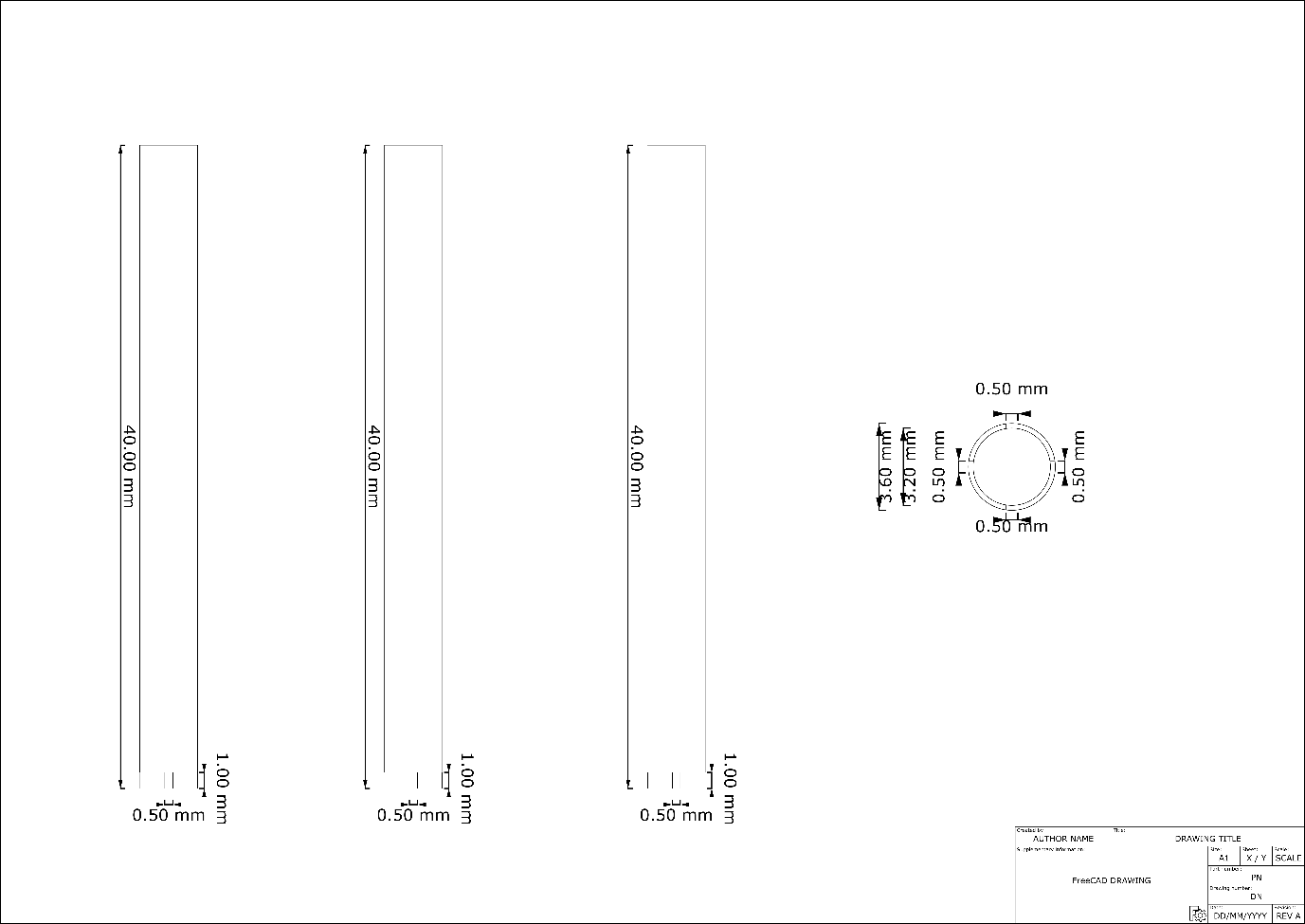


N4 electrode


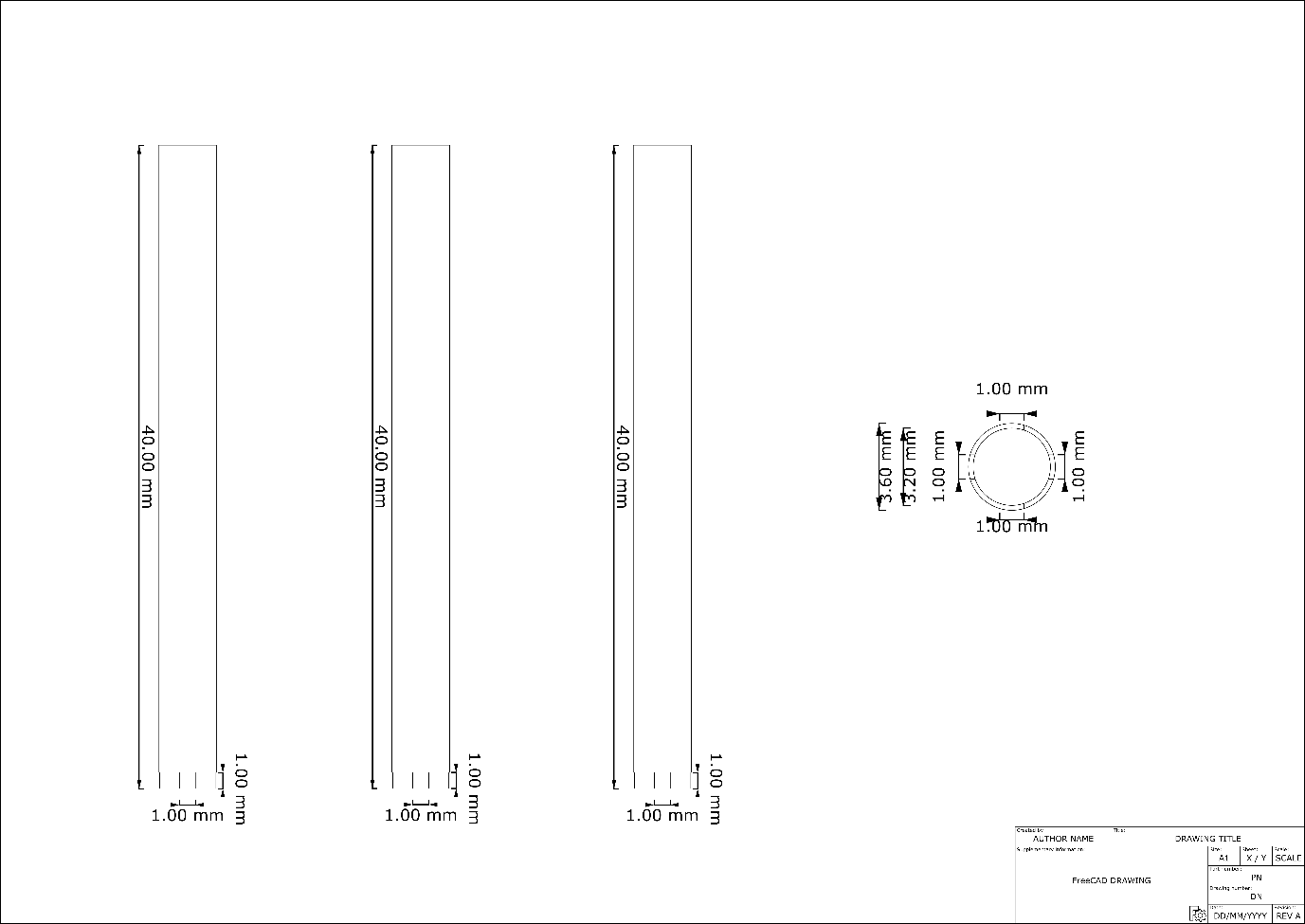


X1 electrode


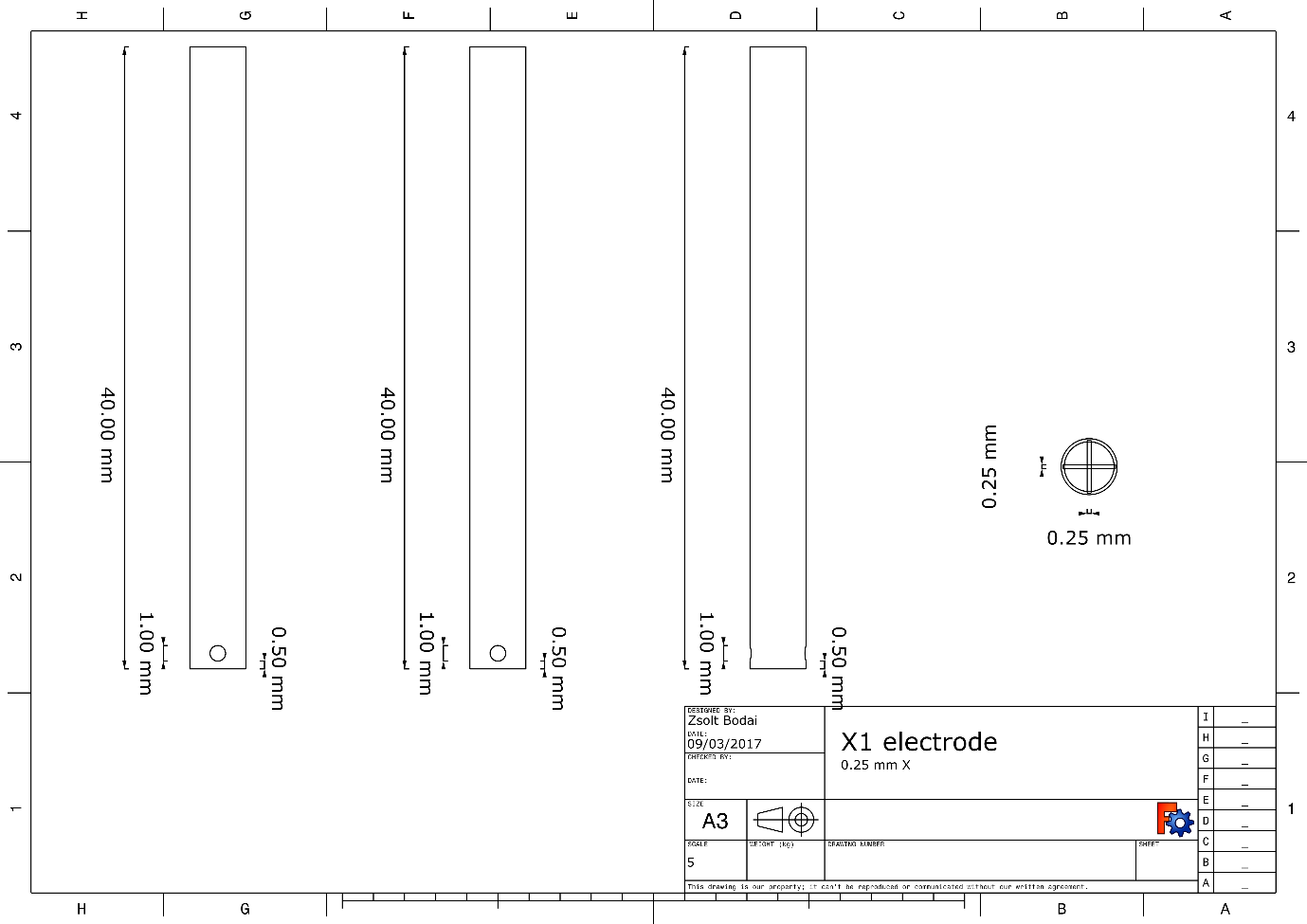


X2 electrode


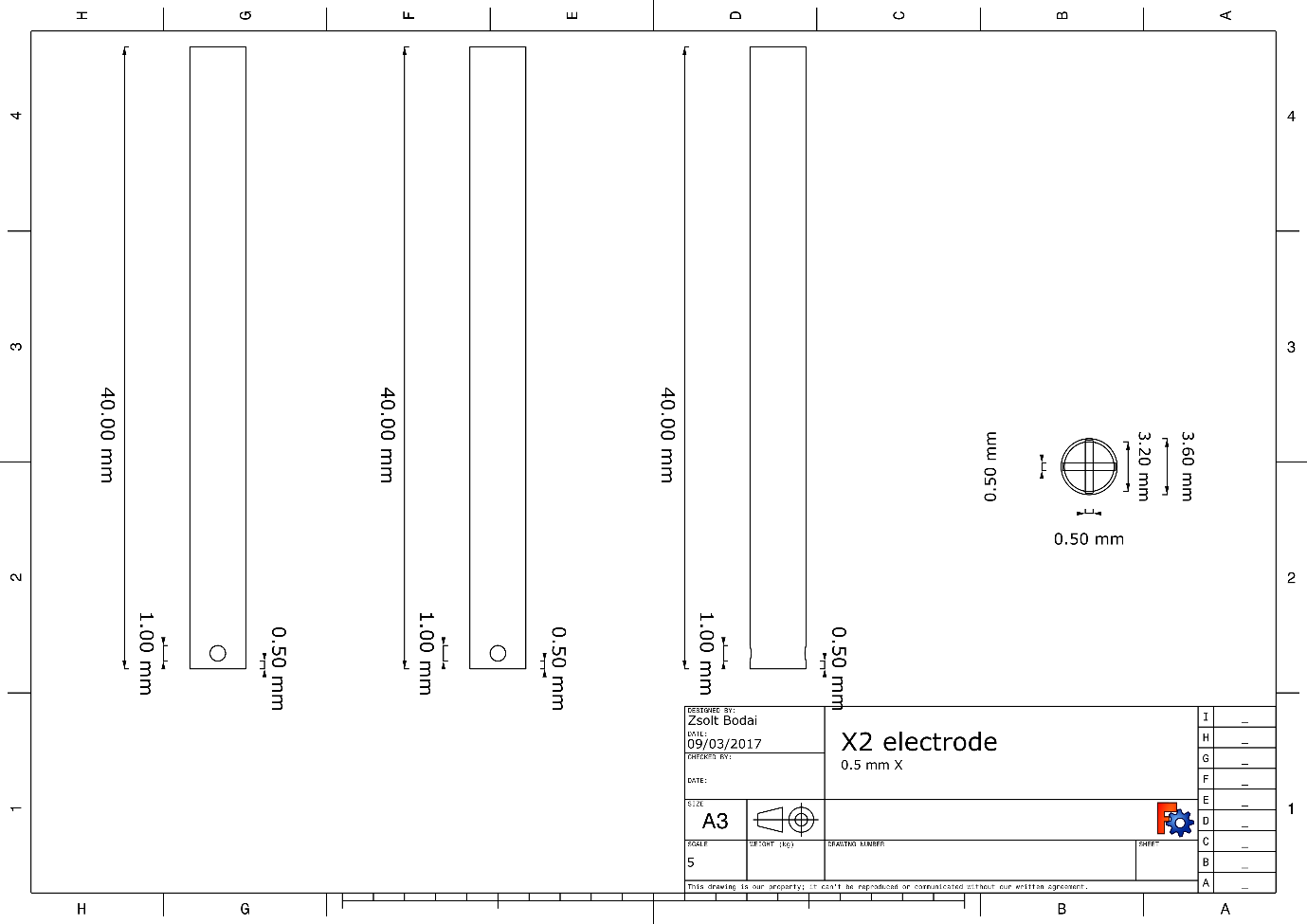


X3 electrode


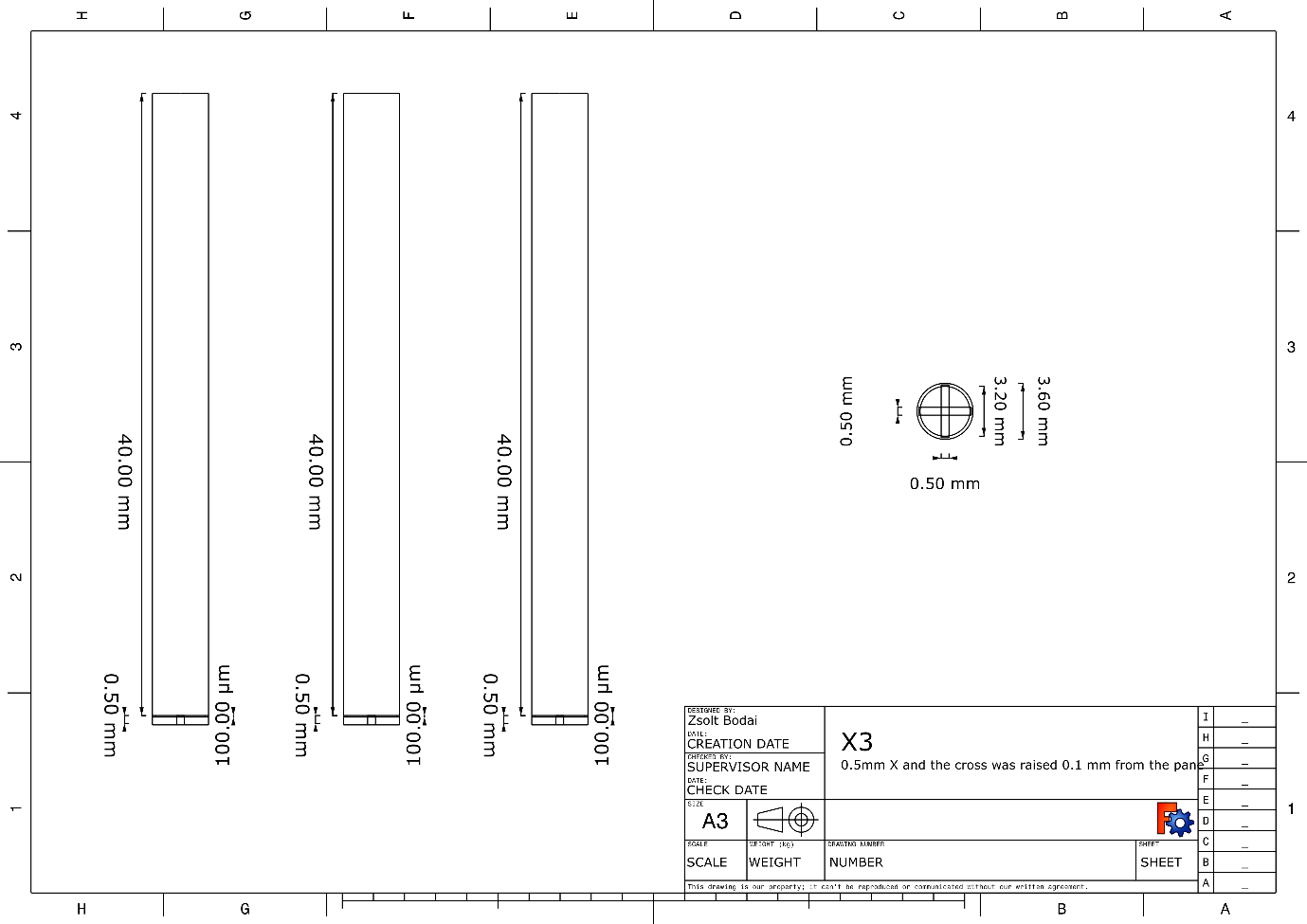


X4 electrode


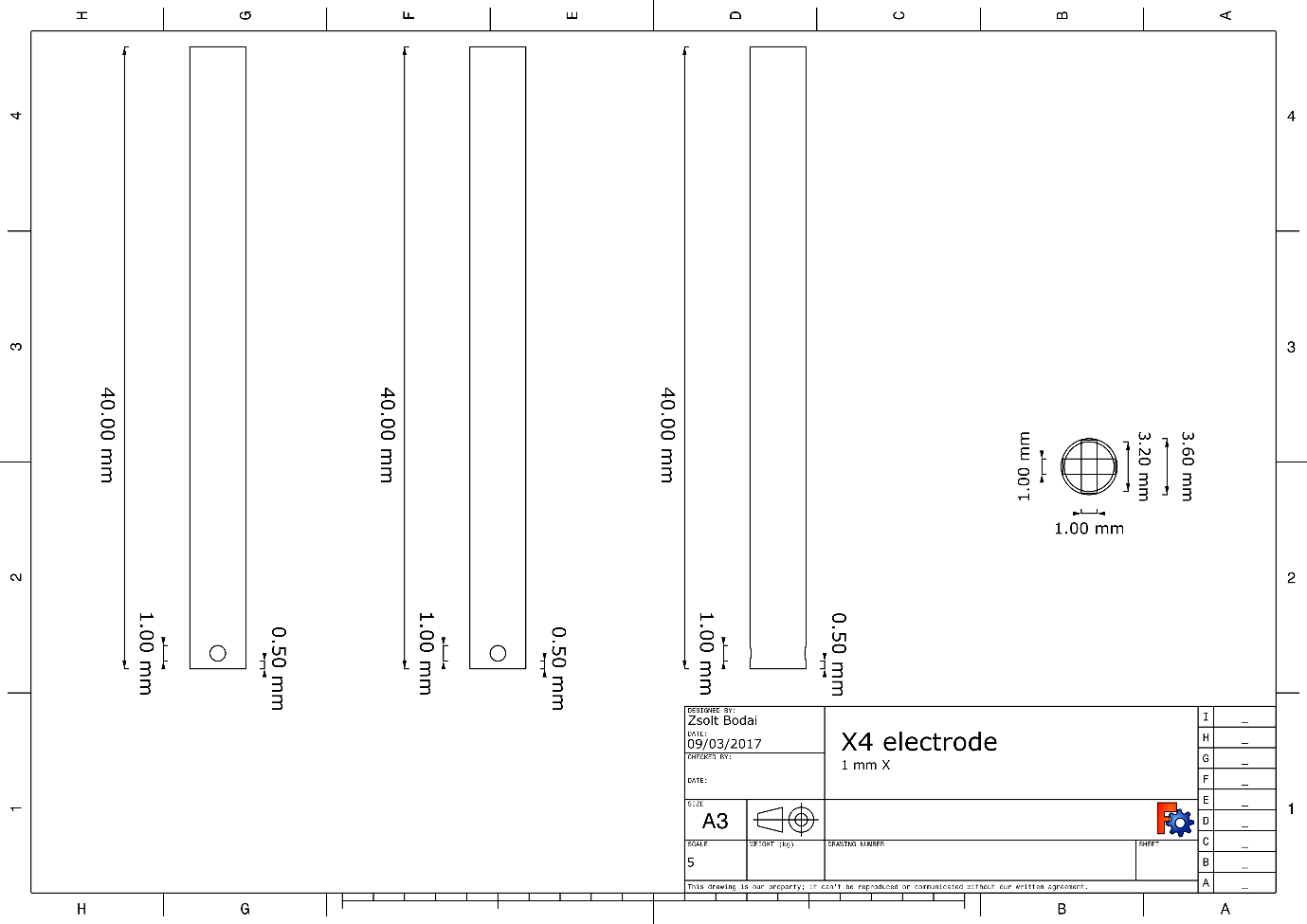


X5 electrode


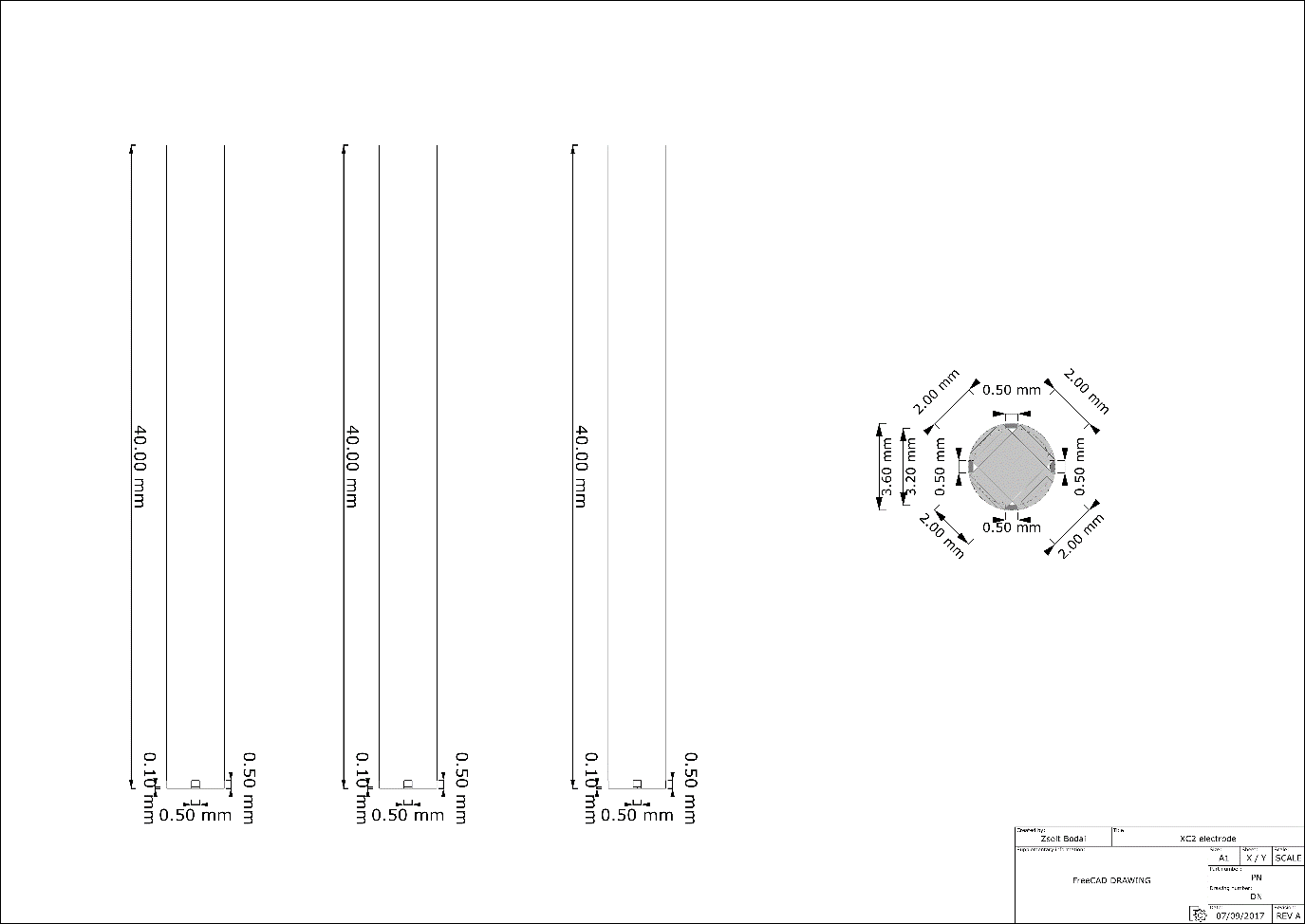


Grey part shows the metal part of the electrode and white the holes where the aerosol can enter the sampling tube

XB electrode


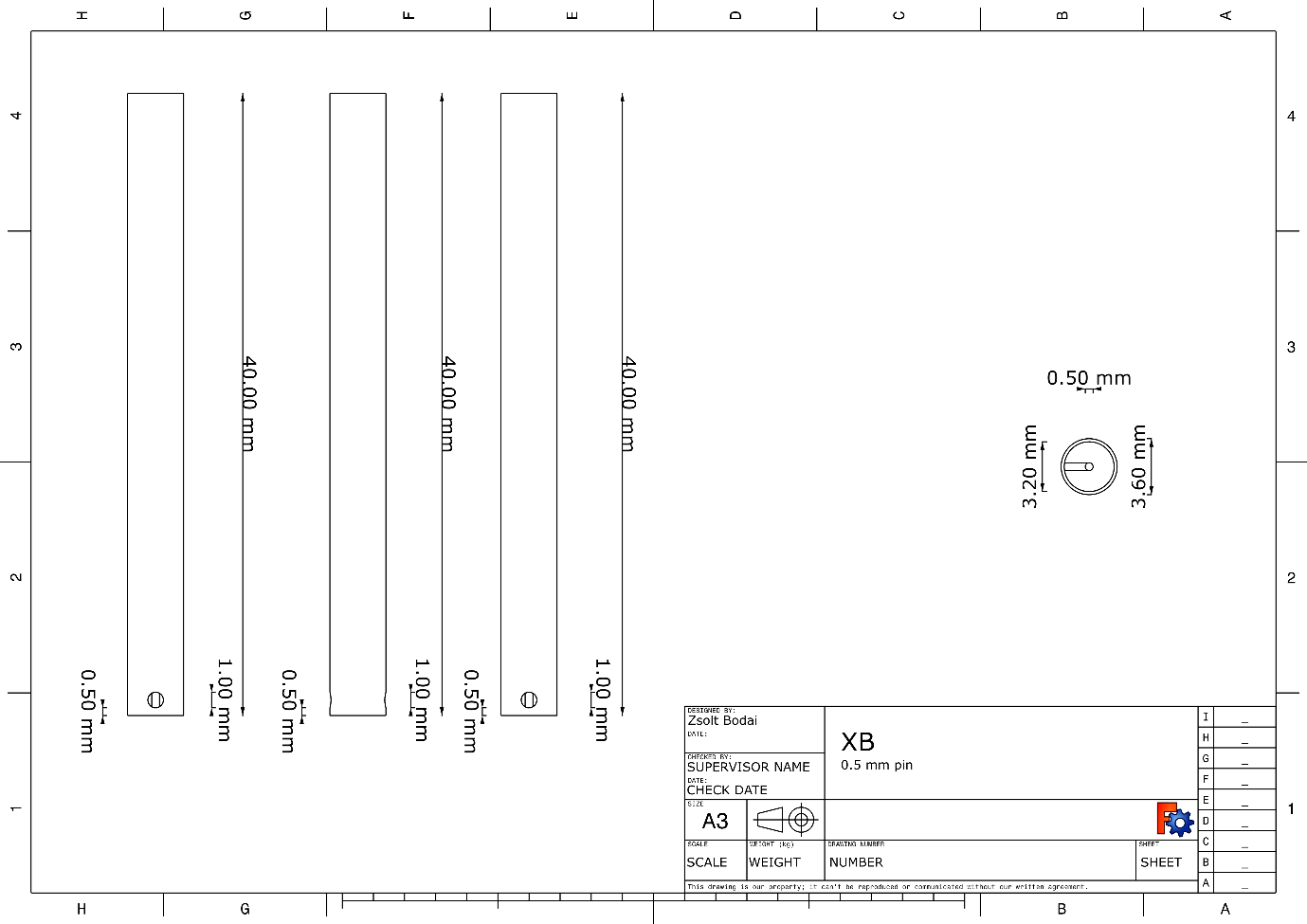


The diameter of the pin is 0.5 mm.

XC1 Electrode


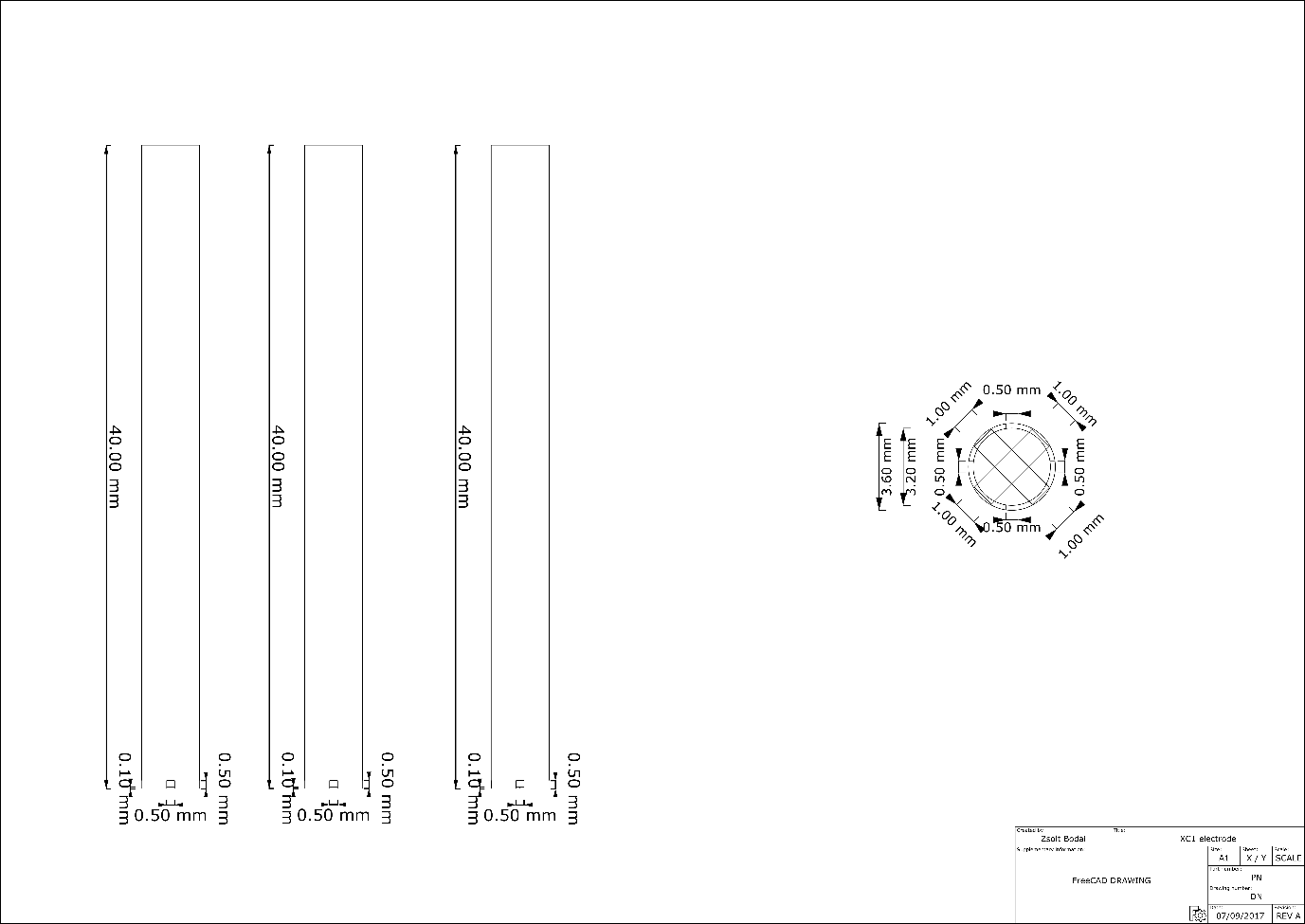


XC2 electrode


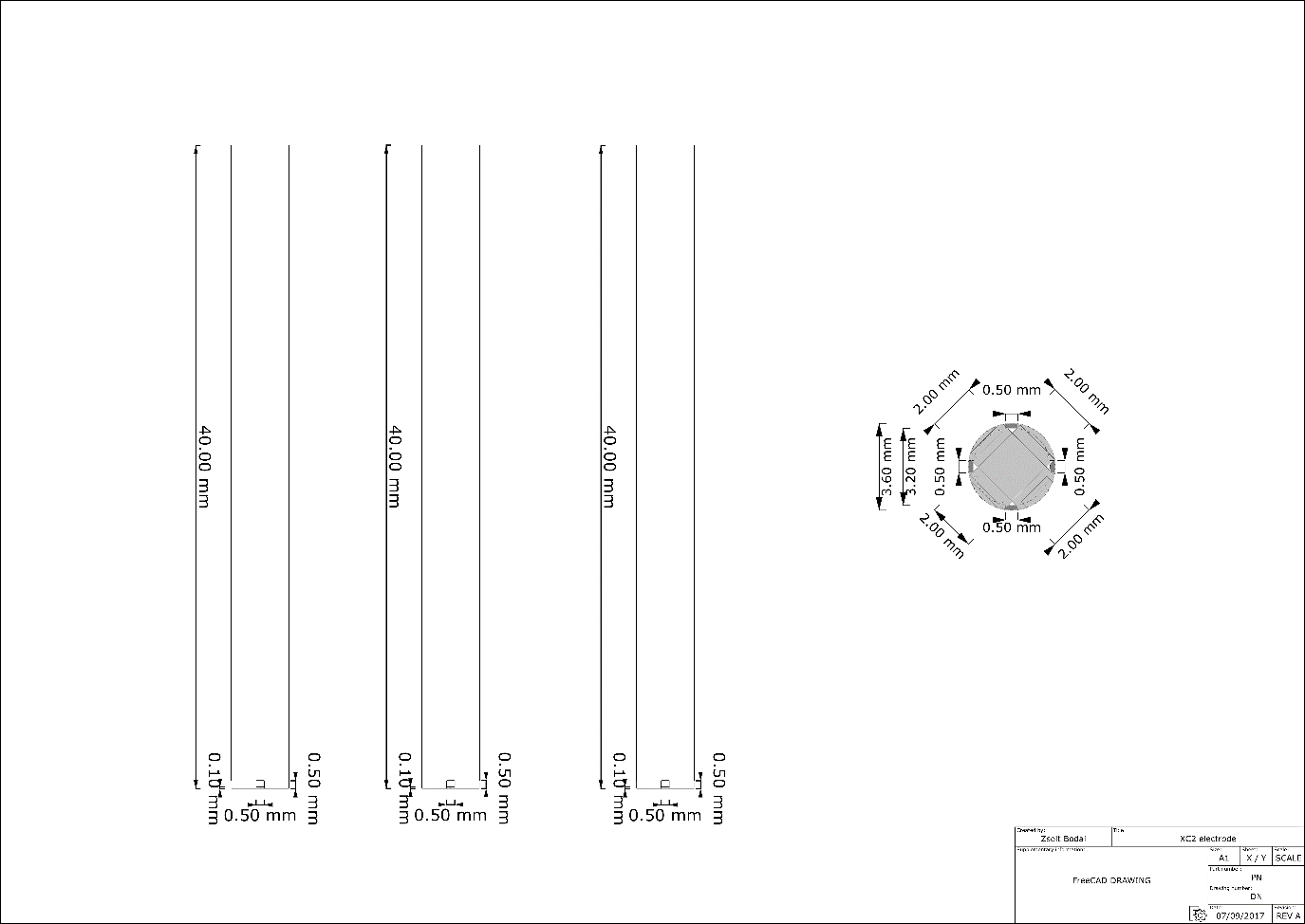


Grey part shows the metal part of the electrode and white the holes where the aerosol can enter the sampling tube

**Electronic supplementary material Figure 3. Absolute intensity, normalised intensity and signal-to-noise ratio for *Escherichia coli, Lactobacillus jensenii* and *Streptococcus pneumoniae*.**

*Escherichia coli*

*Streptococcus pneumonia*

*Lactobacillus jensenii*

TIC normalised intensities, signal-to-noise ratios and absolute intensities are plotted respectively for *Escherichia coli*, *Streptococcus pneumoniae and Lactobacillus jensenii*. Negative ion mode data was exported from OMB to CSV file using background subtraction, lock mass correction and 600-1000 Da range with 0.1 bins as detailed in the paper. For each bacteria one, high intensity peak (bin) was selected to compare the settings. In the case of *Escherichia coli* it was 733.5, *Lactobacillus jensenii* 953.6 and *Streptococcus pneumoniae* 925.6. Background was defined as a summary of the bin intensities between the 600-610 region where could not detect any bacteria related peaks. Signal to noise ratio was calculated for each sampling point using the selected bacteria bin and the summarized background. Error bars on figures represent twice of standard deviation (one up and one down).

Table 1. Classification accuracies with the PCA-LDA leave 20% cross validation and with the MMC leave one plate out cross validation.

| ID | MMC classification accuracy (%) | | PCA-LDA classification accuracy (%) | |
| --- | --- | --- | --- | --- |
|  | negative | positive | negative | positive |
| Original ^a^ | 72 | 65.5 | n.c. | n.c. |
| 3 mm ^b^ | 80 | 66.5 | n.c. | n.c. |
| 2 mm ^b^ | 89 | 90 | n.c. | n.c. |
| 1 mm ^b^ | 89 | 88 | n.c. | n.c. |
| 0.5 mm ^b^ | 86.5 | 87.5 | n.c. | n.c. |
| EL1 | 81.5 | 64 | n.c. | n.c. |
| EL2 | 90.5/91 | 75/87 | 98.5 | 99 |
| EL3 | 88 | 34 | n.c. | n.c. |
| EL4 | 90 | 74.5 | n.c. | n.c. |
| EL5 | 91/87.5 | 84/87 | 99 | 98.5 |
| N1 | 80 | 78 | n.c. | n.c. |
| N2 | 85 | 81 | n.c. | n.c. |
| N3 | 79/91 | 85.5/89.5 | 97.5 | 98.5 |
| N4 | 76 | 77.5 | n.c. | n.c. |
| X1 | 75 | 80.5 | n.c. | n.c. |
| X2 | 81.5/92.5 | 88/85.5 | 99 | 95 |
| X3 ^c^ | 81.5 | 56 | n.c. | n.c. |
| XB | 74 | 79 | n.c. | n.c. |
| X4 | 94 | 80.5 | 98.5 | 97.5 |
| X5 | 91 | 66.5 | 95.0 | 93.5 |
| XC1 | 91 | 80.5 | 97.5 | 97.0 |
| XC2 | 87.5 | 65 | 97.0 | 95.0 |

^a,^ distance between the contact point and the entrance to electrode tubing is 4 mm

^b,^ distance between the contact point and the entrance to electrode tubing

^c,^ the cross was raised 0.1 mm from the pane

n.c. – not calculated
